# Selective Reductive Amination of Carbonyls to Primary Amines Under Ambient Conditions Over Rh/MFM‐300(Cr)

**DOI:** 10.1002/anie.202519641

**Published:** 2025-11-20

**Authors:** Qingqing Mei, Wenyuan Huang, Longfei Lin, Xue Han, Shaojun Xu, Bing An, Svemir Rudić, Rongsheng Cai, Sarah J Haigh, Buxing Han, Martin Schröder, Sihai Yang

**Affiliations:** ^1^ Department of Chemistry The University of Manchester Manchester M13 9PL UK; ^2^ State Key Laboratory of Soil Pollution Control and Safety Zhejiang University Hangzhou 310058 China; ^3^ College of Chemistry and Molecular Engineering Beijing National Laboratory for Molecular Sciences Peking University Beijing 100871 China; ^4^ Beijing National Laboratory for Molecular Sciences CAS Key Laboratory of Colloid Interface and Chemical Thermodynamics Institute of Chemistry Chinese Academy of Science Beijing 100190 China; ^5^ College of Chemistry Beijing Normal University Beijing 100875 China; ^6^ Department of Chemical Engineering University of Manchester Manchester M13 9PL UK; ^7^ ISIS Neutron and Muon Source Rutherford Appleton Laboratory Didcot OX11 0QX UK; ^8^ Department of Materials University of Manchester Manchester M13 9PL UK

**Keywords:** Biomass carbonyls, Heterogeneous catalysis, MOFs, Reductive amination, Rhodium

## Abstract

The synthesis of organic amines via reductive amination of biomass‐derived carbonyl compounds is an important target for sustainable chemical industries. The control of selectivity for the formation of primary amines versus secondary amines is challenging, and high temperature and pressures using H_2_ are required to generate the desired selectivity. Herein, we report the highly selective reductive amination of a broad range of aldehydes and ketones by NH_3_ and H_2_ over Rh/MFM‐300(Cr) to form primary amines with a selectivity of up to 99% under ambient conditions. Inelastic neutron scattering reveals that the Rh species not only promote the hydrogenation process, but also catalyzes the ammonolysis of the Schiff base intermediate, facilitating the selective synthesis of primary amines. This protocol achieves selective reductive amination at 25 °C and 1 atm, providing an energy‐efficient route to a broad spectrum of amines.

## Graphical abstract

Rh/MFM‐300(Cr) enables highly selective reductive amination of biomass‐derived and other carbonyls with NH_3_ and H_2_ to affordprimary amines with up to 99% selectivity under ambient conditions (25 °C, 1 atm).

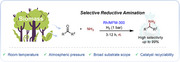

The selective synthesis of organic amines from renewable resources is of central importance for sustainable chemical manufacturing.^[^ ^1^, ^2^, ^3^, ^4^ ^]^ Aldehydes and ketones are valuable organic feedstocks, accessible from abundant lignocellulosic biomass and abundant in natural extracts such as essential oils.^[^ ^5^, ^6^ ^]^ Reductive amination of such renewable carbonyl compounds with NH_3_ and H_2_ offers a sustainable pathway to a wide range of pharmaceuticals, polymers, dyes, and detergents.^[^ ^7^, ^8^, ^9^, ^10^, ^11^, ^12^, ^13^ ^]^ However, the reaction typically yields mixtures of primary and secondary amines, as the primary amines readily undergo further condensation with carbonyls. Enhancing selectivity for primary amines typically demands high concentrations of NH_3_ or pressures (up to 7 bar), yet excess NH_3_ poisons hydrogenation catalysts (Ru, Pt, Co, Ni), necessitating additional H_2_ pressures (≈20 bar) and elevated temperatures to drive the process.^[^ ^14^, ^15^, ^16^, ^17^, ^18^, ^19^ ^]^ Such harsh conditions introduce operational complexity and safety risks. Thus, achieving the synthesis of primary amine from biomass‐derived substrates under mild conditions remains an unresolved challenge, which we address here by developing a process operable at 25 °C and 1 atm.

Metal–organic frameworks (MOFs) provide unique opportunities to integrate active sites at defined positions within confined pore environments, thus enabling the control of reaction pathways.^[^ ^20^, ^21^, ^22^, ^23^, ^24^, ^25^, ^26^ ^]^ MOF catalysts have shown promise in diverse applications including water splitting,^[^ ^27^ ^]^ photo‐conversion of CO_2_ and CH_4_^[^ ^28^, ^29^ ^]^ as well as organic transformations.^[^ ^22^ ^]^ Yet their application in reductive amination with NH_3_/H_2_ has not been realized, owing to their limited stability in NH_3_ at elevated temperatures and the challenge of selectively controlling C─O and C─N bond transformations in complex reaction systems. We have recently developed the robust MFM‐300 family of materials, which exhibits reversible NH_3_ adsorption over many cycles and enables efficient activation of aldehydes and ketones at ambient temperature through strong host–guest hydrogen bonding in confined pores.^[^ ^30^, ^31^ ^]^ These features establish a unique platform for reductive amination of carbonyl compounds under mild conditions.

Herein, we report the exceptional performance of MFM‐300(Cr) as a support for Rh nanoparticles [Rh/MFM‐300(Cr)] in the reductive amination of diverse biomass‐derived carbonyl compounds with NH_3_ and H_2_ under ambient conditions (25 °C, 1 atm). Primary amines are obtained with selectivities of up to 99%. Inelastic neutron scattering and control experiments reveal that Rh sites not only promote hydrogenation, but also catalyze the ammonolysis of Schiff base intermediates, thereby steering the reaction pathway towards primary amines.

Cyclohexanone was employed as a model substrate to test for reductive amination with NH_3_ (3.5 M in MeOH) and H_2_ (1 bar, 25 °C, 3 h) (Table S2). Among the catalysts that were tested, Rh/MFM‐300(Cr) delivered full conversion with 98% selectivity to cyclohexanamine. Activity depended strongly on the method of preparation of the catalyst: NaBH_4_‐reduced Rh/MFM‐300(Cr) was highly active, whereas H_2_‐reduction or double‐solvent encapsulation^[^ ^32^ ^]^ rendered it inactive, indicating that surface‐exposed Rh sites are essential. The catalyst also tolerated diluted H_2_ (30% in Ar) and variable NH_3_ concentrations without loss of selectivity, allowing direct use of Haber‐process gas mixtures. A yield of 92% was maintained over 10 cycles (Figure S1), which established Rh/MFM‐300(Cr) as a highly active and durable catalyst for reductive amination under ambient conditions.

A wide range of ketones and aldehydes were converted to primary amines using Rh/MFM‐300(Cr) (Figure 1). Both aromatic and aliphatic aldehydes afforded high yields (71%–99%). Notably, while conjugated C═C bonds were hydrogenated (**P22**), isolated double bonds remained intact (**P15, P18**). Interestingly, ketones show higher activity though they are generally more difficult to reduce than aldehydes, affording the corresponding branched primary amines with high selectivity. This behavior can be rationalized by the rapid and reversible formation of trimeric hemiaminal adducts from aldehydes with NH_3_, which markedly decreases the concentration of catalytically‐active free carbonyl species.^[^ ^33^, ^34^ ^]^ In contrast, ketones are less prone to such association due to steric and electronic factors, rendering them more accessible for reductive amination. Given their abundance in nature and accessibility from biomass, we also examined natural products and biomass‐derived carbonyls, achieving consistently high yields of primary amines. For example, levulinic acid (**P17**) can be converted quantitatively to 5‐methyl‐2‐pyrrolidinone under ambient conditions, rivaling the best catalysts reported.^[^ ^35^ ^]^ This system also enables the efficient synthesis of pharmaceutically relevant molecules. Amphetamines (**P28‐P30**), a family of central nervous system stimulants, can be obtained in high yields under ambient conditions. Amantadine (**P27**), a key therapeutic for Parkinson's disease and a reported antiviral for COVID‐19,^[^ ^36^ ^]^ was also produced quantitatively. These results underscore the broad utility of Rh/MFM‐300(Cr) in the reductive amination of diverse carbonyl compounds under ambient conditions. A comparison with reported systems shows that this system stands among the leading catalysts for selective reductive amination of biomass‐derived carbonyls to primary amines under ambient conditions (Table S1).

**Figure 1:**
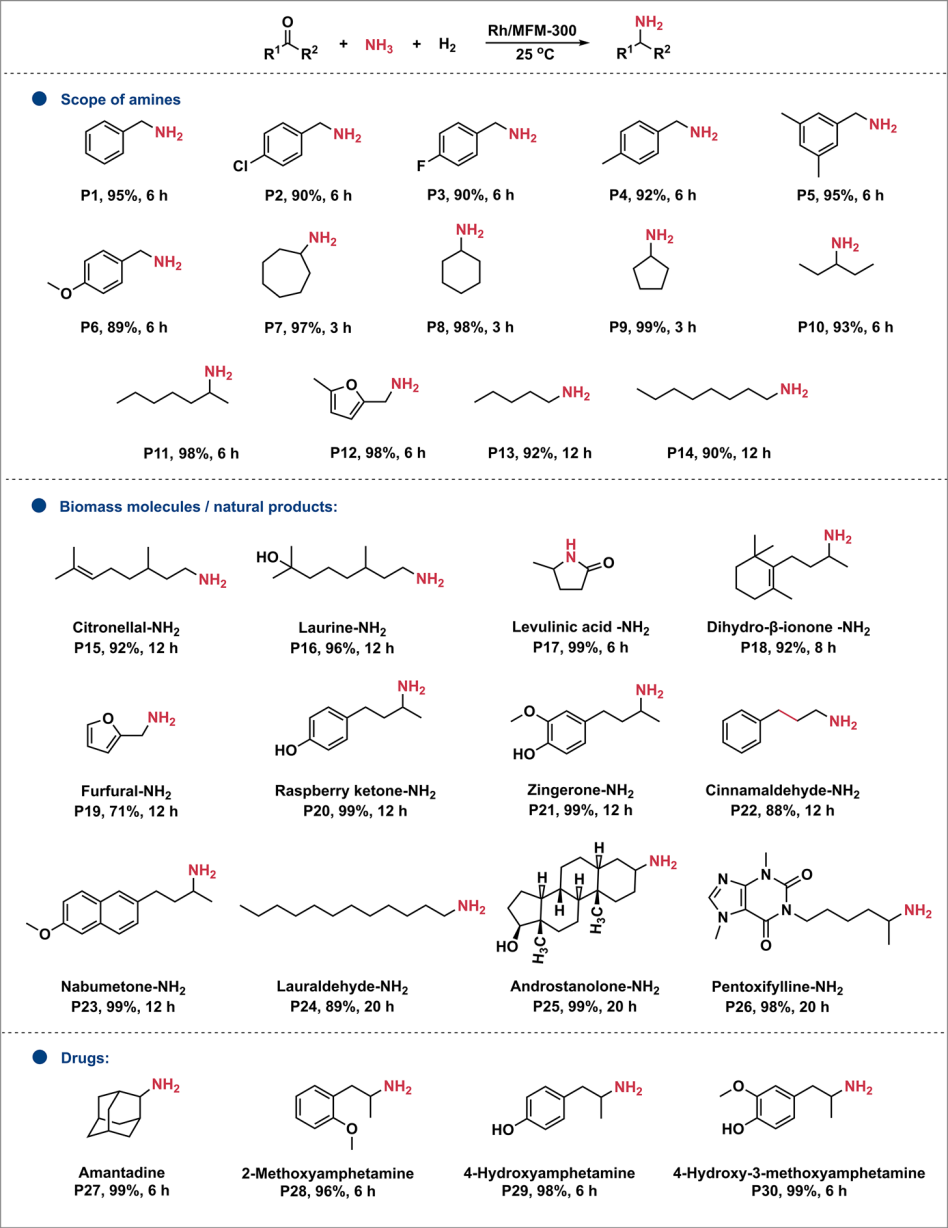
Scope of the reductive amination reaction. Reaction conditions: Rh/MFM‐300 (20 mg, 0.0039 mmol), substrate (1.0 mmol), 3.5 mol L^−1^ NH_3_/MeOH solution (4 mL), H_2_ (1 bar), 25 °C, 3∼20 h. (new Fig 1 attached)

To understand the structural origin of this activity, we performed detailed characterization of the catalyst. The powder X‐ray diffraction (PXRD) patterns of fresh and used Rh/MFM‐300(Cr) catalysts confirm retention of crystal structure of MFM‐300(Cr) and absence of bulk Rh and Rh_2_O_3_ (Figure 2b).^[^ ^37^, ^38^, ^39^ ^]^ High‐angle annular dark‐field scanning transmission electron microscopy (HAADF STEM) images of Rh/MFM‐300(Cr) show that the rod‐like MOF crystallites are decorated with Rh nanoparticles (average diameter of ∼2.2 nm) on the external surface (Figure 2a). After 10 catalytic cycles, only slight particle growth to ∼2.7 nm is observed (Figure S2). Porosity was determined by N_2_ adsorption and 77 K. Although the BET surface area of Rh/MFM‐300(Cr) drops from 1136 to 528 m^2^ g^−1^ upon loading of Rh, the average pore size remained mostly unchanged (Figure S3) consistent with the Rh nanoparticles residing mainly on the external surface of MFM‐300(Cr), as confirmed by HAADF STEM experiments (Figure 2b).

**Figure 2:**
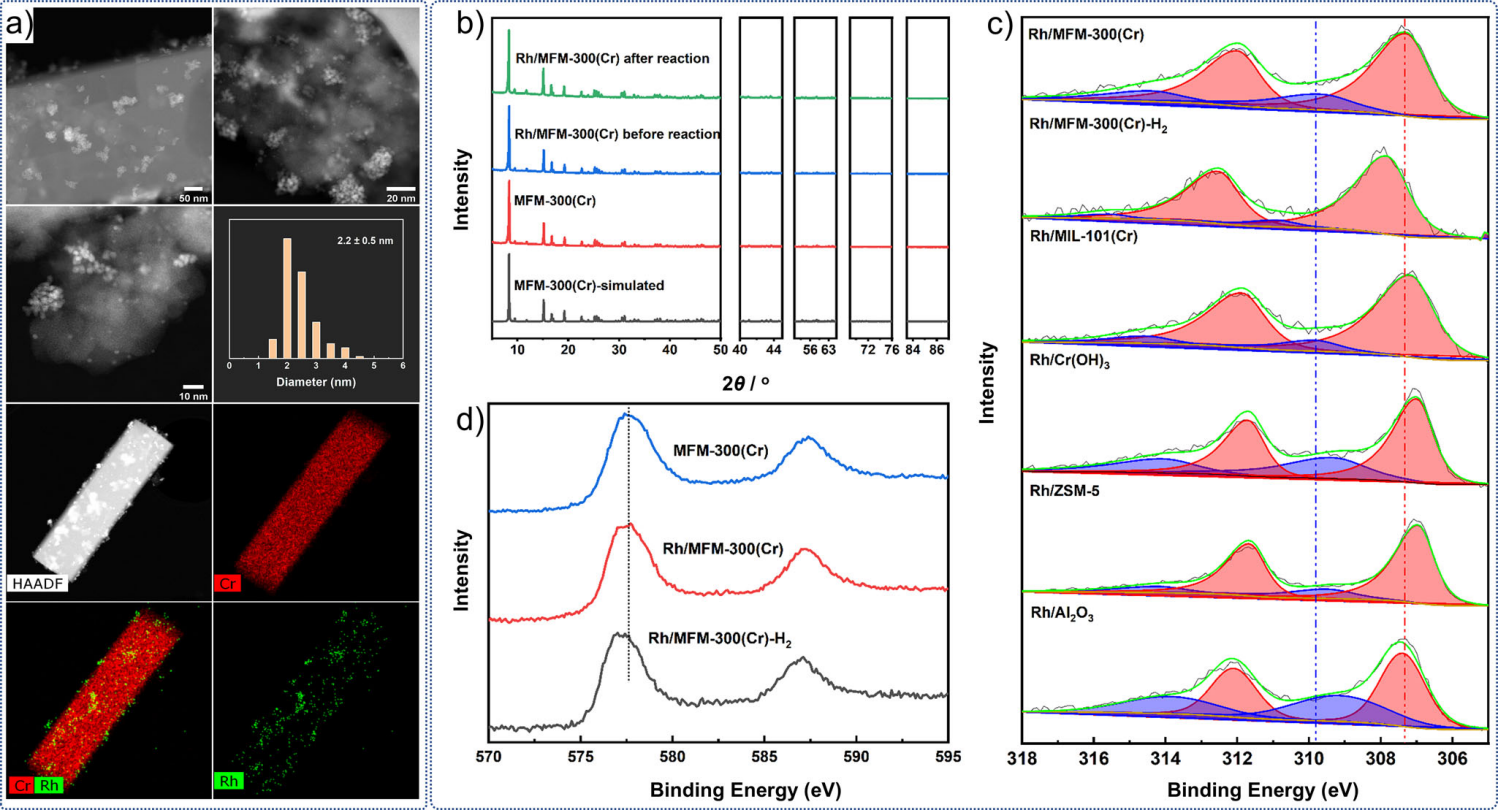
Characterization of the Rh/MFM‐300(Cr) catalyst. a) HAADF STEM images and energy dispersive X‐ray spectroscopy (EDS) elemental mapping; b) PXRD patterns of the catalyst before and after reaction to confirm the absence of bulk Rh; c) and d) XPS profile of Rh3d and Cr2p in different catalysts.

The surface electronic states of Rh were probed by X‐ray photoelectron spectroscopy (XPS). The Rh 3d spectrum of Rh/MFM‐300(Cr) displays the characteristic 3d_5/2_ and 3d_3/2_ spin‐orbit peaks, which can be deconvoluted into two doublets, indicating the coexistence of Rh(0) and Rh(III) states (Figure 2c). The binding energy of Rh 3d_5/2_ of the fitted curves are 307.3 and 309.7 eV, and are attributed to Rh(0) and Rh(III), respectively;^[^ ^40^, ^41^ ^]^ the former is 0.2 eV higher than that of bulk Rh (307.1 eV),^[^ ^40^ ^]^ indicating the Rh(0) species in Rh/MFM‐300(Cr) are electron‐deficient. The Rh 3d peaks for 2 wt% Rh/MIL‐101(Cr) appear at nearly identical positions as Rh/MFM‐300(Cr), consistent with their similar catalytic performance. Although the Rh(0) 3d peak of Rh/Al_2_O_3_ is close to Rh/MFM‐300(Cr), the Rh(III) 3d peak shifts to lower binding energy (BE), which likely corresponds to the lower activity of Rh/Al_2_O_3_. Similarly, the diminished Rh(III) content in H_2_‐reduced Rh/MFM‐300(Cr)‐H_2_ correlates with its lower performance, underscoring the importance of Rh(III) in promoting reductive amination. The Cr2p peaks of Rh@MFM‐300(Cr)‐H_2_ shift to lower BE (Figure 2d), revealing a charge transfer from Rh to MFM‐300(Cr) in the H_2_‐reduced sample. In contrast, the Cr2p peak position of Rh/MFM‐300 (Cr) is the same as in pristine MFM‐300(Cr), indicating the absence of charge transfer from Rh to the MOF, analogous to observations for Pt@MIL‐101(Cr).^[^ ^22^ ^]^

To further elucidate the local coordination environment of Rh, X‐ray absorption fine structure (XAFS) analysis of Rh/MFM‐300(Cr) at the Rh K‐edge was performed. In the near‐edge region (Figure 3a), the white‐line intensity of the X‐ray absorption near‐edge structure (XANES) spectra of Rh/MFM‐300(Cr) resides between that of Rh foil and Rh_2_O_3_, corroborating the coexistence of Rh(0) and Rh(III) species. Fourier‐transformed k^2^‐weighted extended X‐ray absorption fine structure (EXAFS) in R space shows two peaks at 1.6 and 2.5 Å (Figure 3b), attributable to Rh─O and Rh─Rh scattering, respectively.^[^ ^42^ ^]^ Compared with the reference materials (Rh foil and Rh_2_O_3_), the diminished intensity of long‐range features suggests small well‐dispersed Rh nanoparticles, consistent with TEM observations.

**Figure 3:**
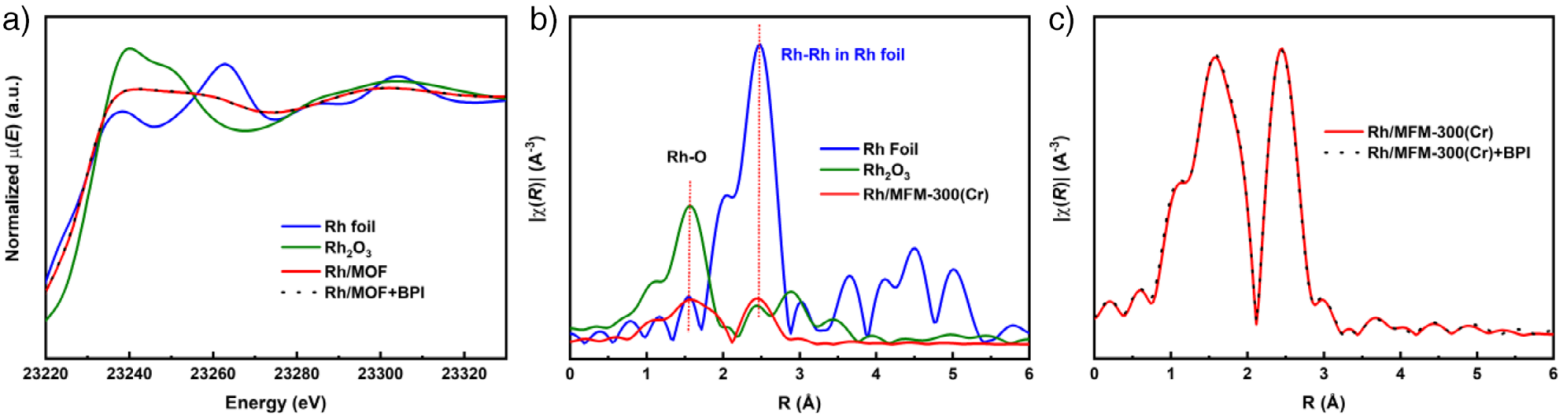
XAS characterization of the Rh/MFM‐300(Cr) catalyst. a) The normalized X‐ray absorption near‐edge spectra (XANES) at the Rh K‐edge of the catalyst and reference materials; b) The k^2^‐weighted Fourier transform extended X‐ray absorption fine structure spectra (EXAFS) of different catalysts in R‐space; c) EXAFS of catalyst with and without adsorption of BPI.

Control experiments (Table S2) using catalysts with distinct Rh oxidation states and configurations further reveal that while Rh(0) species predominantly mediate H_2_ activation and hydrogenation, their electronic structure and consequently their activity are fine‐tuned by adjacent Rh(III) sites through interfacial electronic modulation. Additionally, the Rh(III) sites also act as Lewis acid centers to activate carbonyl groups and facilitate imine formation. This cooperative interplay between Rh(0) and Rh(III) parallels the oxidation‐state synergy observed in Ru/RuO_2_ systems.^[^ ^11^, ^43^, ^44^ ^]^

We next investigated the reaction mechanism of this catalysis. Kinetic studies confirm that the substrate is transformed rapidly to the corresponding Schiff base,^[^ ^45^ ^]^ which is subsequently converted to amine (Figures S4 and S5), indicating that transformation of the Schiff base intermediate is the rate‐determining step. The Schiff base can be hydrogenated directly to secondary amine or transformed to imine or gem‐diamine to yield primary amines upon hydrogenation. Thus, the evolution of the Schiff base determines the selectivity of products (Figure S6). To probe this pathway, ^15^ *N*‐benzyl‐1‐phenylmethanimine (BPI), the intermediate in the reductive amination of benzaldehyde, was employed as a representative Schiff base substrate. ^15^N NMR spectroscopy confirms that BPI remains unreacted in the absence of catalyst (Figure S7), highlighting the role of Rh/MFM‐300(Cr) in promoting its transformation to benzylamine. The interaction between Rh/MFM‐300(Cr) and BPI was studied by X‐ray absorption spectroscopy (XAS). In the near‐edge region (Figure 3c), the spectrum of the BPI‐loaded catalyst is identical to that of the unloaded catalyst, and the EXAFS analysis in R space also shows negligible differences, indicating minimal interaction between Rh sites and the Schiff base. Thus, the Schiff base is not activated directly at the Rh center, effectively preventing the over‐hydrogenation to secondary amines.

To directly probe unstable intermediates, we employed *operando* inelastic neutron scattering (INS), a powerful tool for monitoring vibrational dynamics.^[^ ^46^ ^]^ Although imines and gem‐diamines are widely proposed intermediates (Figure 4a), their detection has been limited by their instability (Figure 4a). Here, INS afforded direct visualization of their adsorption states to de‐convolute the reaction pathways. BPI was adsorbed onto Rh/MFM‐300(Cr), and, to better monitor reaction intermediates, NH_3_ and H_2_ were dosed separately. Dosing NH_3_ onto BPI@Rh/MFM‐300(Cr) generates the corresponding gem‐diamine, which was observed by INS. H_2_ was then dosed in the presence of NH_3_, and the gem‐diamine was hydrogenated to give the primary amine. Overall, the INS spectrum of adsorbed BPI is similar to that of bulk BPI, and the DFT‐simulated INS spectrum of BPI shows excellent agreement with the experimental data (Figure 4c). The peaks at low energy transfer (below 150 cm^−1^), assigned to the lattice modes of BPI, decrease significantly upon adsorption, suggesting hindered motions due to the strong adsorption onto the catalyst. The intensities of peaks at 1026 cm^−1^ (C_7_─N stretching), 1208 cm^−1^ (C_7_─H twisting), and 1376 (C_9_─H rocking) cm^−1^ also decrease suggesting interaction of the C═N bond with the catalyst.

**Figure 4:**
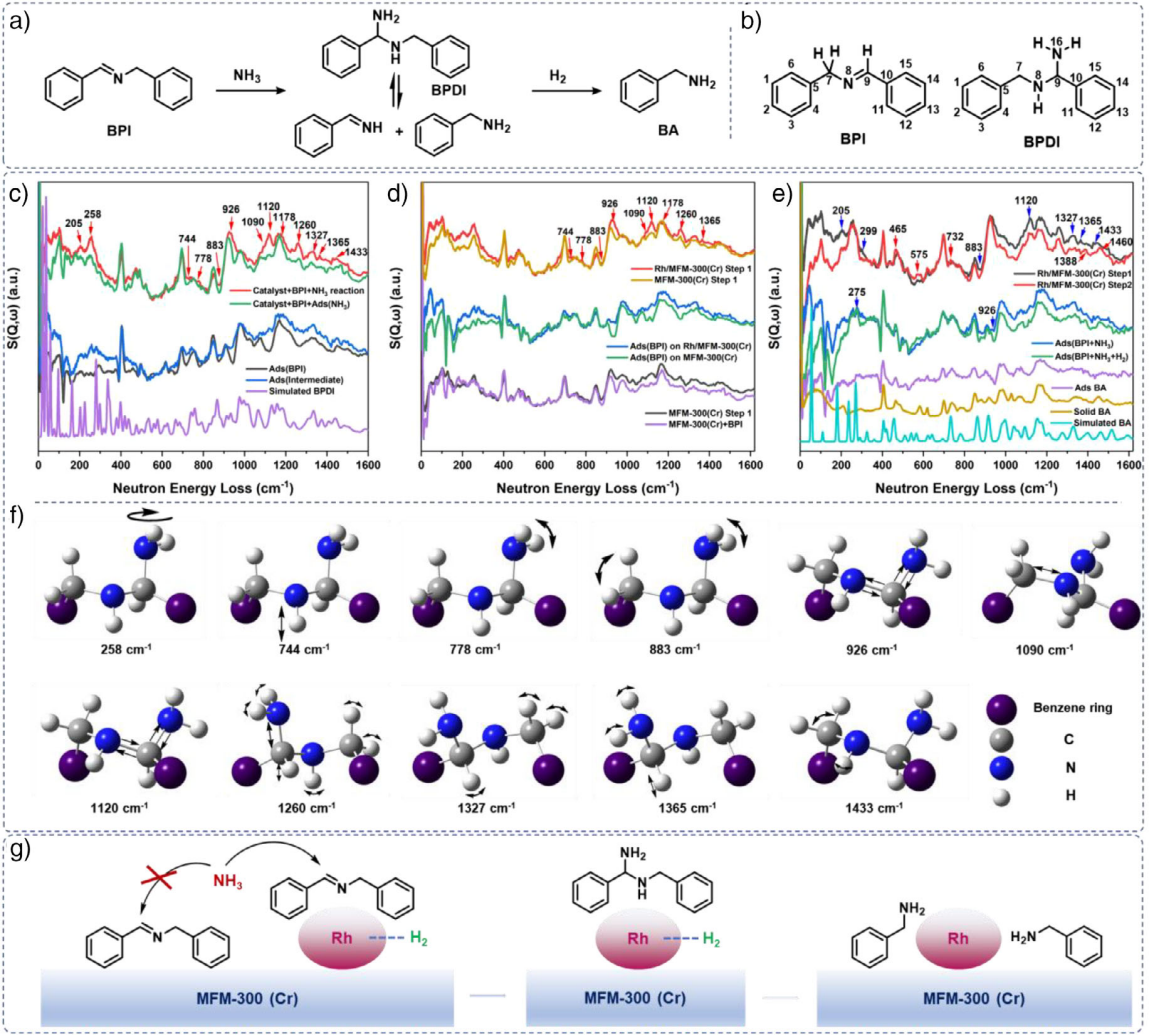
INS study of the reaction mechanism. a) Transformation of Schiff base to primary amine; b) labeling of atoms; c) INS spectra of adsorbed BPI and adsorbed intermediate after reaction with NH_3_: Ads (X) is the signal of adsorbed X after subtraction of the signal due to catalyst and cell; d) comparison of INS spectra after the reaction of adsorbed BPI with NH_3_ (step 1) on Rh/MFM‐300(Cr); e) INS spectra after reaction with NH_3_ (step 1) and reaction with NH_3_ and H_2_ (step 2); f) assigned vibrational modes; g) reactivity of the Schiff base on the surface of the catalyst.

The reaction of adsorbed BPI with NH_3_ was conducted at 25 °C for 2 h to monitor the possible formation of unstable intermediates such as *N*‐benzyl‐1‐phenylmethanediamine (BPDI). On reaction with NH_3_, the INS peaks (Figure 4c) changed significantly compared to that of adsorbed BPI, and the new spectrum shows good agreement with the DFT‐simulated spectrum of BPDI. A notable new peak appears at 1120 cm^−1^, assigned to the asymmetric stretching vibration of the N_8_‐C_9_‐N_16_ moiety, and the peak corresponding to the symmetric stretching at 926 cm^−1^ increases in intensity. The C─N stretching vibration is observed at 1090 cm^−1^ (C_7_─N_8_, C_9_─N_8_, C_9_─N_16_ stretching), and N─H vibrations are located at 258 cm^−1^ (─NH_2_ torsion), 744 cm^−1^ (N_8_─H rocking), 778 cm^−1^ (N_16_─H wagging), 883 cm^−1^ (N_16_─H wagging coupled with C_7_─H rocking), 1178 cm^−1^ (N_16_─H twisting coupled with C_9_─H rocking) and 1260 cm^−1^ (N_16_─H twisting coupled with C_7_─H twisting). All these peaks match closely with the simulated spectrum of BPDI, thus confirming its formation. Interestingly, the peak due C_9_─H rocking (1376 cm^−1^), which disappears on adsorption of DPI, is regenerated (1365 cm^−1^) upon reaction with NH_3_. We postulate that on conversion of BPI to BPDI via addition of NH_3_ to the C═N bond, the C_9_─H rocking is no longer restricted due to C═N adsorption onto the catalyst. The peak due to C─C─C twisting of the benzene ring (205 cm^−1^) is also enhanced due to the consumption of the C═N bond. The sample was then exposed to H_2_ (3 bar) at room temperature for 2 h (Figure 4e). The peaks for BPDI at 883, 926, 1120, 1327 and 1365 cm^−1^ decreased, indicating their consumption, and new peaks at 465, 575, and 732 cm^−1^ corresponding to C─C out‐of‐plane wagging, C─H twisting and C─H wagging in the benzene ring, respectively, are observed. The C─C─C twisting motions at 205 and 299 cm^−1^ (coupled with ─NH_2_ torsion) also decrease suggesting that the environment of the benzene ring changes. Shifts in peaks from 258 to 275 cm^−1^ (─NH_2_ torsion), 1365 to1388 cm^−1^ (C─H wagging coupled with NH_2_ twisting), and 1433 to 1460 cm^−1^ (C─H scissoring) imply the formation of a new species, and overall, the peaks are consistent with the INS spectrum of benzylamine. The formation of benzylamine was confirmed further by online mass spectrometry.

We also studied this reaction by INS using pristine MFM‐300(Cr) following the same procedures as above. Unlike Rh/MFM‐300(Cr), no peaks corresponding to BPDI were generated with MFM‐300(Cr) apart from a peak at 258 cm^−1^ assigned to the ─NH_2_ torsional twist due to NH_3_ interacting with BPI via hydrogen bonding (Figure 4d). This indicates that the key addition step does not occur on MFM‐300(Cr) alone in the absence of Rh. Accordingly, the catalytic pathway from BPI to benzylamine can be established (Figure 4g): BPI adsorbs onto Rh sites, where it undergoes addition with NH_3_ to form BPDI; subsequently, active H species on Rh facilitate the hydrogenation of BPDI to benzylamine under mild conditions. These findings provide direct spectroscopic evidence for the debated initial NH_3_ addition step in reductive amination catalysis.^[^ ^45^ ^]^

In summary, we have developed a highly selective catalytic system that promotes the reductive amination of aldehydes and ketones to primary amines at room temperature and atmospheric pressure. A broad range of primary amines, including pharmaceutical molecules, can be synthesized under ambient conditions with high selectivity (up to 99%). XPS and XAS analyzes demonstrate the crucial role of both Rh(0) and Rh(III) species, and INS/DFT analysis reveals that the Rh species not only promote the hydrogenation process, but also directly participate in ammonolysis of the Schiff base intermediate. These insights establish a new design principle for multifunctional catalysts to enable the sustainable synthesis of amine‐based chemicals.

## Conflict of Interests

The authors declare no conflict of interest.

## Supporting information



Supporting Information

## Data Availability

The data that support the findings of this study are available in the Supporting Information of this article.
