# Supplementary material for: Selective Reductive Amination of Carbonyls to Primary Amines Under Ambient Conditions Over Rh/MFM‐300(Cr)
Source: Angew Chem Int Ed Engl. 2025 Nov 20;64(52):e19641. doi: 10.1002/anie.202519641 (PMC12723456; doi:10.1002/anie.202519641)

**Supplementary Information**

**Selective Reductive Amination of Carbonyls to Primary Amines under Ambient Conditions over Rh/MFM-300(Cr)**

Qingqing Mei,^[a,b]^ Wenyuan Huang,^[a,c]^ Longfei Lin,^[d]^ Xue Han,^[e]^ Shaojun Xu,^[f]^ Bing An,^[a]^ Svemir Rudić,^[g]^ Rongsheng Cai,^[h]^ Sarah J. Haigh,^[h]^ Buxing Han,^[d]^ Martin Schröder^[a]^* and Sihai Yang^[a,c]^*

a. Department of Chemistry, The University of Manchester, Manchester, M13 9PL, UK

b. State Key Laboratory of Soil Pollution Control and Safety, Zhejiang University, Hangzhou 310058, China

c. College of Chemistry and Molecular Engineering, Beijing National Laboratory for Molecular Sciences, Peking University, Beijing, 100871, China

d. Beijing National Laboratory for Molecular Sciences, CAS Key Laboratory of Colloid, Interface and Chemical Thermodynamics, Institute of Chemistry, Chinese Academy of Science, Beijing, 100190, China

e. College of Chemistry, Beijing Normal University, Beijing, 100875, China

f. Department of Chemical Engineering, University of Manchester, Manchester, M13 9PL, UK

g. ISIS Neutron and Muon Source, Rutherford Appleton Laboratory, Didcot, OX11 0QX, UK

h. Department of Materials, University of Manchester, Manchester, M13 9PL, UK

**Contents**

[1. Methods 3](#_Toc211372770)

[2. Supplementary Figures 5](#_Toc211372771)

[2.1 Recycle test 5](#_Toc211372773)

[2.2 N_2_ sorption isotherms and pore size distribution 7](#_Toc211372774)

[2.3 Kinetic study 8](#_Toc211372775)

[2.4 Reaction of the Schiff base 10](#_Toc211372776)

[2.5 ^15^N NMR spectroscopic analysis 11](#_Toc211372777)

[3. Supplementary Tables 12](#_Toc211372778)

[3.1 Reported data for state-of-the-art catalysts 12](#_Toc211372772)

[3.2 Reductive amination over various catalysts 13](#_Toc211372779)

[3.3 Effect of NH_3_ and H_2_ pressure 16](#_Toc211372780)

[3.4 Assignment of vibrational modes in INS 17](#_Toc211372781)

[4. References 20](#_Toc211372782)

[5. NMR spectra of substrates 22](#_Toc211372783)

1. Methods

**Preparation of catalyst.**

**Synthesis of MFM-300(Cr):** CrCl_3_·6H_2_O (200 mg, 0.751 mmol) and biphenyl-3,3’,5,5’- tetracarboxylic acid (H_4_L) (70.0 mg, 0.212 mmol) were added to a mixture of H_2_O (10 mL) and HCl (1%, 1.5 mL) and the suspension stirred at room temperature for 30 min. The solution was transferred to a 23mL Teflon autoclave and heated at 210 °C for 3 days. After cooling to room temperature, the blue microcrystalline solid was collected via filtration, washed with H_2_O (20 mL × 2) and acetone (20 mL × 2) and dried in air (yield: 87%).

**Preparation of Rh/MFM-300 (Cr):** In a typical experiment, MFM-300(Cr) (0.5g) and RhCl₃·3H₂O (0.026g) were dispersed in MeOH (10mL) and sonicated for 30 mins. The solution was transferred into an ice bath and stirred vigorously for another 30 mins. NaBH_4_ (0.05g) was dissolved in an NH_3_/MeOH solution (4 mL, 3.5mol/L) and placed in an ice bath for 10 mins. This was then added to the MOF mixture dropwise with vigorous stirring for 2 h. The catalyst was collected by centrifugation and washed with MeOH several times and dried under vacuum at room temperature for 24 h. The content of Rh was determined to be 2.09% by ICP-MS measurements. Other catalysts, including Pt/MFM-300 (Cr), Pd/MFM-300 (Cr), Ru/MFM-300 (Cr), and Rh catalysts with different supports were prepared by the same method.

**Storage stability:** The as-prepared Rh/MFM-300(Cr) catalyst was sealed and stored under a N_2_ atmosphere to prevent adverse effects of air and moisture on its structure and activity. The catalyst can retain stable physicochemical properties and catalytic performance for at least one month.

**Catalytic reaction.** In a typical run, 1.0 mmol cyclohexanone and 20 mg catalyst were added to a 500 mL Schlenk flask under N_2_. H_2_ gas was introduced to the flask and NH_3_/MeOH solution (8 mL 3.5mol/L) was injected into the flask, and a H_2_ balloon connected. The reactor was placed in a 25^o^C water bath for the desired reaction time. After reaction, the mixture was centrifuged, and the liquid phase was collected for quantitative analysis using Gas Chromatography (GC) and NMR.

**Catalyst recovery:** After each reaction, the reaction mixture was centrifuged. The solid Rh/MFM-300(Cr) catalyst remaining in the centrifuge tube was subsequently washed three times with MeOH (10 mL each time) and then dried at room temperature.

**Characterisation.** PXRD data were collected on an X-ray powder diffractometer (Phillips X’pert MPD) with Cu-Kα radiation at a scan speed of 1.5^o^/min. Elemental analysis for C, H and N content of MFM-300 (Cr) were carried out using a CE-440 Elemental Analyser manufactured by Exeter Analytical. STEM characterization was performed using a Thermo Fisher Talos F200A microscope equipped with a Schottky field emission gun (FEG) and a Super-X energy-dispersive X-ray spectroscopy (EDS) system. ICP-MS measurements for analysis of Cr and Rh were carried out using a PerkinElmer NexION 300X ICP-MS (PerkinElmer, USA). ^1^H NMR and ^13^C NMR spectra were measured on Bruker B500 or B400 spectrometers. X-ray absorption spectra (XAS) at Rh K-edge were measured on beamline B18 at the Diamond Light Source, Didcot, UK. N_2_ sorption isotherms were measured on a Micromeritics 3-Flex gas sorption analyser at 77 K. Samples were activated at 170°C for 36 h under vacuum before measurement.

**Inelastic neutron scattering (INS) experiments**. INS spectra were recorded on TOSCA at the ISIS Facility at the STFC Rutherford Appleton Laboratory (UK). All the INS spectra were collected after the sample was cooled and stabilized at temperatures below 15 K. The adsorption/reaction experiments were conducted at the TOSCA beamlines. In a typical experiment, the catalyst (~4 g) was loaded into a cell and heated at 60 °C under He for 24 h to remove any remaining trace water before the experiment. BPI (0.9 g) was injected into the cell at 25 °C. Before the data collection, the cell was flushed using dry He to remove weakly bound BPI. The samples were cooled to <15 K before data collection. After each reaction, the cell was quenched in liquid N_2_ for INS collection to detect the presence of possible reaction intermediates. INS spectra of starting material and reaction products were collected at 5 K. To better monitor reaction intermediates, NH_3_ and H_2_ were dosed separately. In the first step, NH_3_ was dosed to generate the gem-diamine intermediate, which was detected by INS. In the second step, H_2_ was dosed in the presence of NH_3_, and the gem-diamine intermediate was hydrogenated to the primary amine. DFT calculations were performed using Gaussian 16 package^[1]^ to calculate the vibration frequency of BPI, BPDI and BA at B3LYP/6-311+g** level, and the INS spectra was generated using the OCLIMAX program.^[2]^ The simulated spectra was used to identify the modes of vibrational features in the experimental INS spectra.^[3]^

2. Supplementary Figures

2.1 Recycle test


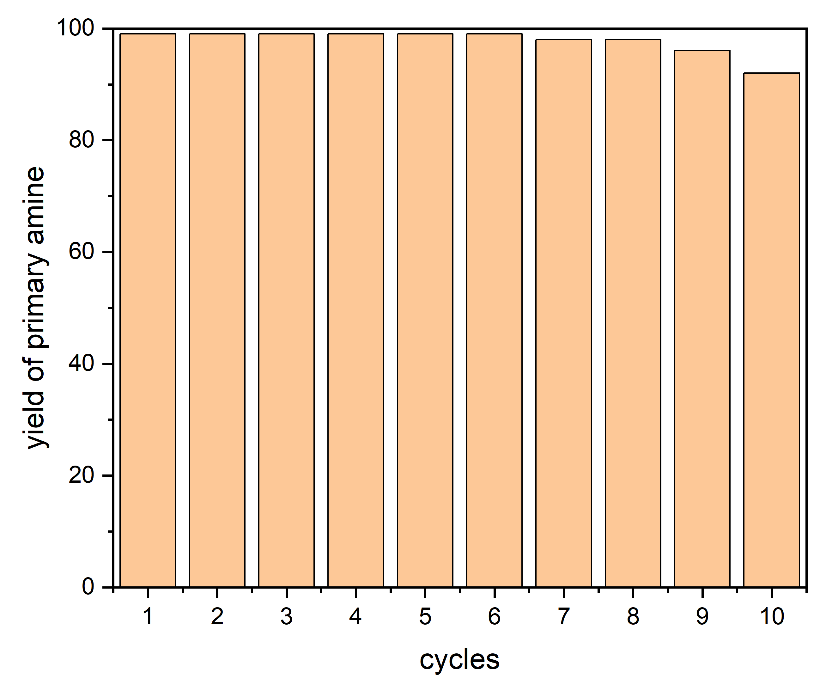


**Fig. S1**. The recycle test of Rh/MFM-300(Cr) for reductive amination of cyclohexanone. Each run was conducted for 6 h under ambient conditions.


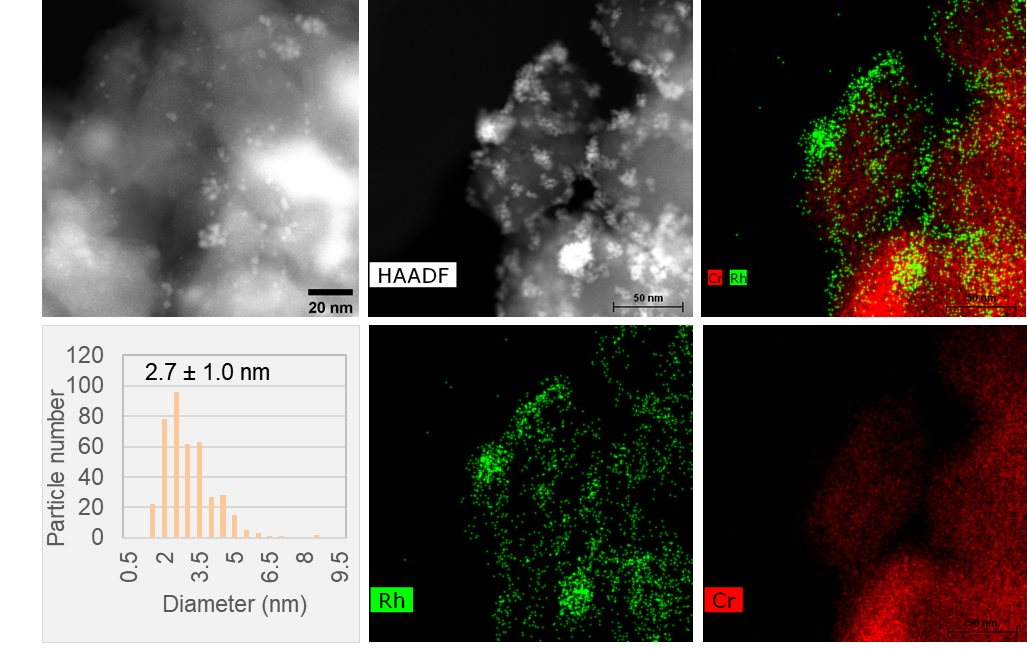


**Fig. S2**. STEM analysis of Rh nanoparticle agglomeration after 10 times repeated use.

As shown in Figure S2, after ten catalytic cycles, the rod-like MOF crystallites were partially fractured into irregular shapes. However, the Rh nanoparticles remained uniformly dispersed on the MOF surface, similar to the fresh sample. The average particle size increased slightly from 2.2 to 2.7 nm. Despite this change, the catalyst maintained a high yield of ~92% after ten consecutive cycles, suggesting that the primary active Rh sites remained immobilized with the MFM-300(Cr) framework.

2.2 N_2_ sorption isotherms and pore size distribution


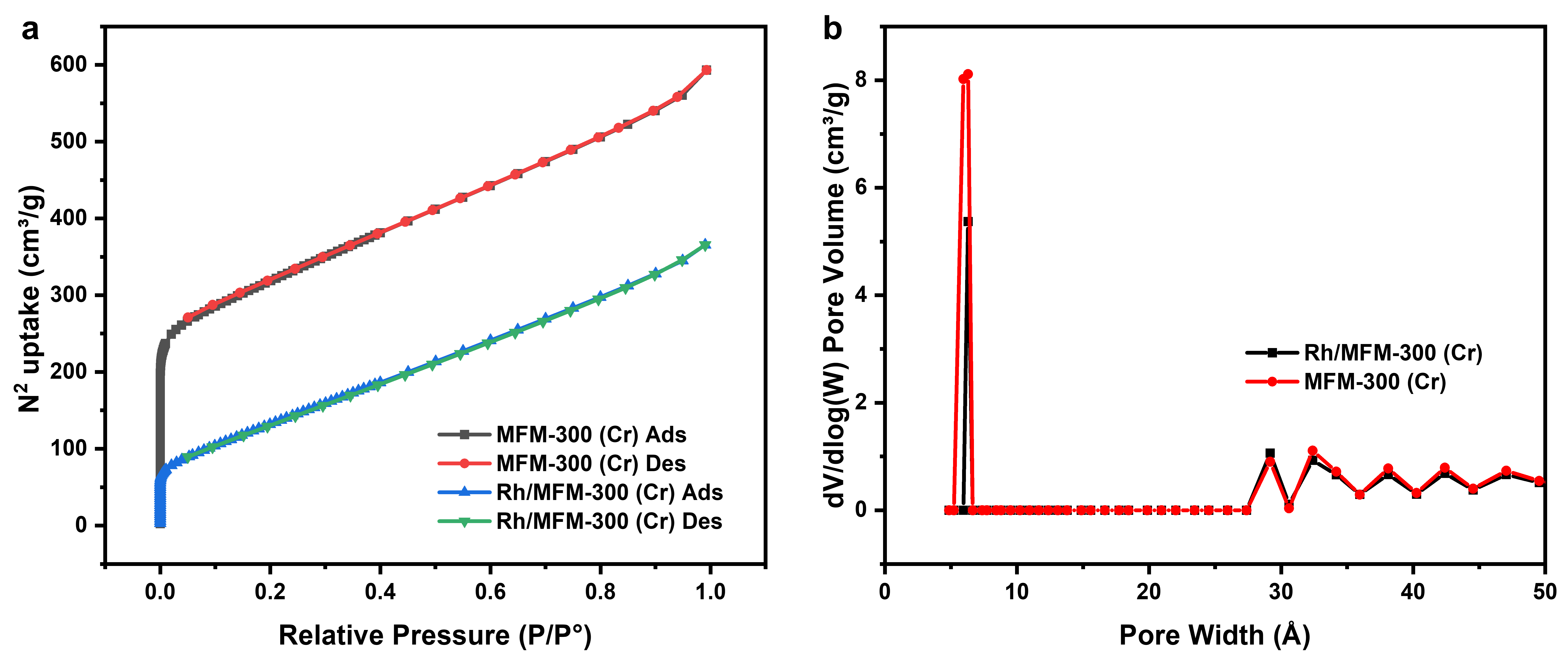


| Samples | BET surface area  (m^2^/g) | Average pore size  (nm) |
| --- | --- | --- |
| MFM-300 (Cr) | 1136 | 4.3 |
| Rh/MFM-300 (Cr) | 528 | 4.2 |

**Fig. S3**. (a) N_2_ adsorption and desorption isotherms for MFM-300(Cr) and Rh/MFM-300(Cr) at 77K. (b) Pore size distribution for MFM-300(Cr) and Rh/MFM-300(Cr) calculated using the Horvath-Kawazoe method.

The value of ~4.2 nm corresponds to interparticle mesopores generated from the packing and aggregation of MFM-300(Cr) crystallites, rather than the intrinsic micropores of the framework. The nearly unchanged mesopore size (4.3 → 4.2 nm), together with TEM and EDX elemental mapping, supports that Rh nanoparticles are predominantly (but perhaps not entirely) deposited on the external surface, rather than blocking the pores of MFM-300(Cr).

2.3 Kinetic study

**
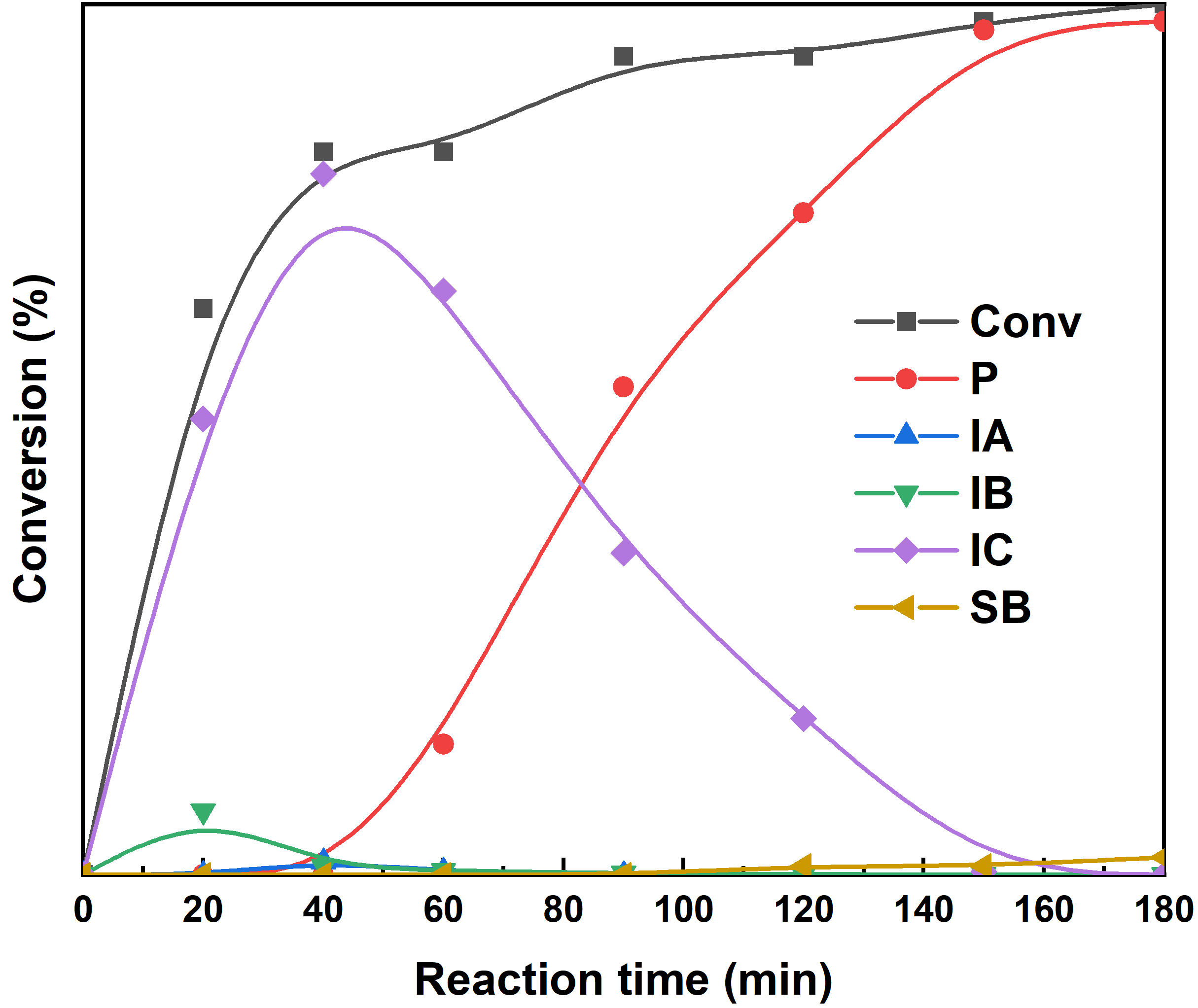
**

**Fig. S4.** Evolution of species during the reaction as a function of time.

**
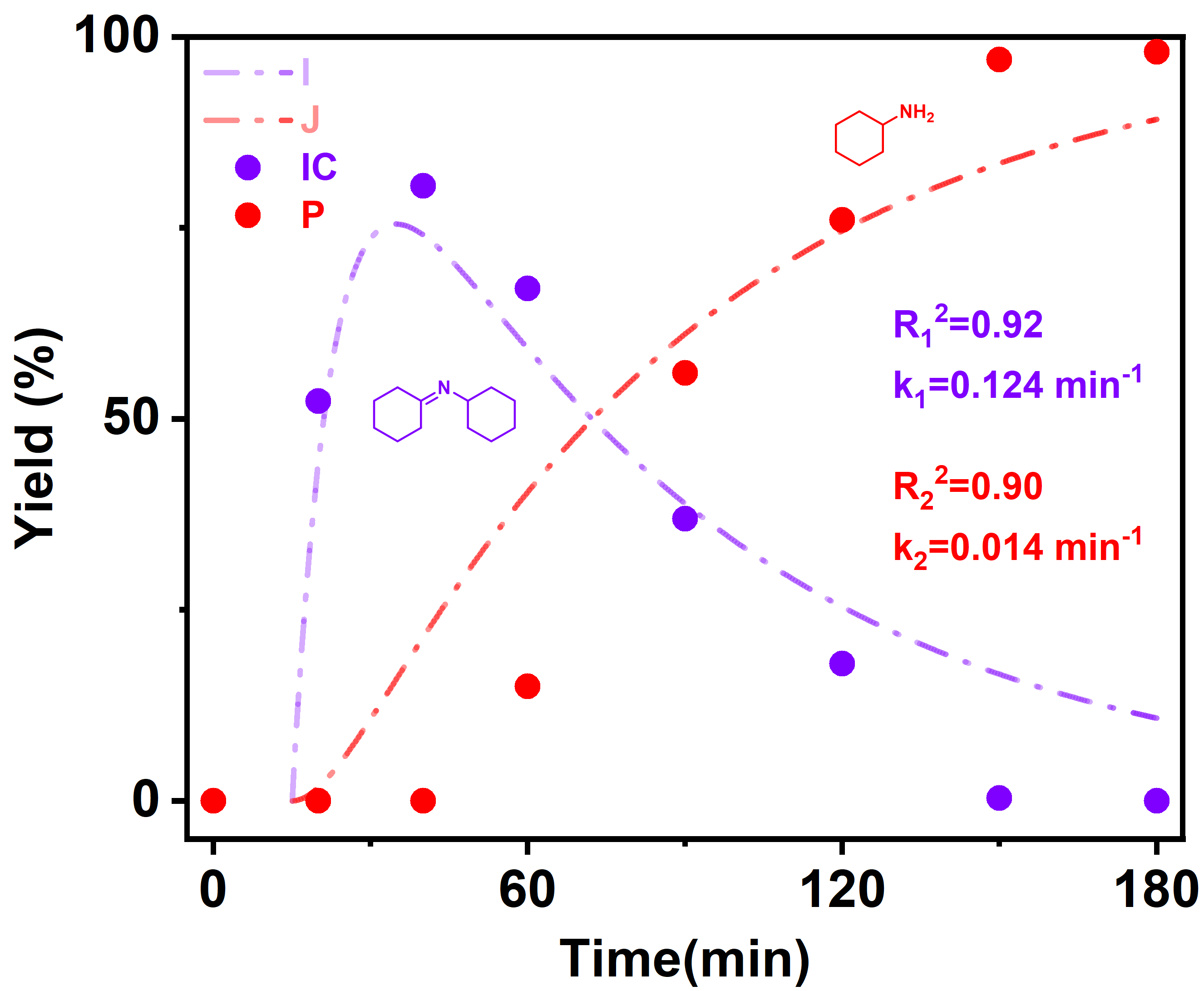
**

**Fig. S5.** Kinetic fitting based on a consecutive reaction model (**R** → **IC** → **P**).

In this model, **IC** represents the Schiff base intermediate and **P** denotes the final product. The rate constants k₁ (**R** → **IC**) and k₂ (**IC** → **P**) were obtained by numerical fitting of the experimental time-course data under strict physical constraints (A₀ fixed to 1). This gave the best-fit parameters *k_1_* = 0.124 min^-1^ for **R**→**IC** and *k_2_* = 0.0142 min^-1^ for **IC**→**P**. These results indicate that the formation of the Schiff base intermediate is relatively fast, while its subsequent conversion to the final product is slower, confirming that the transformation of the Schiff base is the rate-determining step.

2.4 Reaction of the Schiff base

**Fig. S6.** Reaction of Schiff base intermediate to produce primary and secondary amines.

2.5 ^15^N NMR spectroscopic analysis

**a**

**b**

**Fig. S7.** ^15^N NMR spectra of ^15^N labelled BPI (a) before and (b) after treatment in NH_3_/Methanol solution under reaction conditions without catalyst.

3. Supplementary Tables

3.1 Reported data for state-of-the-art catalysts

**Table S1. Products of reductive animation of benzaldehyde reported in the literature**

| Entry | Catalyst | Temperature/℃ | Pressure/bar | time | Yield of benzylamine | References |
| --- | --- | --- | --- | --- | --- | --- |
| 1 | Fe_2_PNC/ZrO_2_ | 150 | 37 | 3 | 94 | [4] |
| 2 | Pt-MoO_x_/TiO_2_ | 150 | 6 | 20 | 9 | [5] |
| 3 | [Rh(cod)Cl]_2_ + TPPTS | 135 | >65 | 2 | 86 | [6] |
| 4 | Fe/(N)SiC | 130 | 65 | 20 | 89 | [7] |
| 5 | RhCl_2_(PPh_3_)_3_ | 130 | 45 | 24 | 95 | [8] |
| 6 | [Ru(CO)ClH(PPh_3_)_3_] + DPPE | 120 | 44 | 16 | 95 | [9] |
| 7 | Ni-TA@SiO_2_-800 | 120 | 20 | 24 | 88 | [10] |
| 8 | Co-DABCO-TPA@C-800 | 120 | 45 | 15 | 87 | [11] |
| 9 | Co/Gs@C | 120 | 45 | 15 | 87 | [12] |
| 10 | Fe@HC700 | 110 | 20 | 4 | 92.2 | [13] |
| 11 | Co2P nanorods | 100 | 5 | 10 | 94 | [14] |
| 12 | Co(BF_4_)_2_.6H_2_O+ Triphos | 100 | 45 | 24 | 97 | [15] |
| 13 | Ru_1_/NC-900-800NH_3_ | 100 | 25 | 10 | 97 | [16] |
| 14 | PtMo nanowire | 100 | 1 | 24 | 0 | [17] |
| 15 | Ru/HAP | 100 | >3 | 2 | 32 | [18] |
| 16 | Ni@C-2 | 100 | 4 | 6 | 98 | [19] |
| 17 | Ru/C | 90 | >40 | 5.3 | 98 | [20] |
| 18 | Pd/C | 90 | >40 | 2.6 | 65 | [21] |
| 19 | Ru/TiO_2_-200A-H | 90 | 10 | 2 | 94.2 | [22] |
| 20 | Ru/SBA-15 | 90 | 40 | 5 | 95 | [23] |
| 21 | RuNP | 90 | 20 | 4 | 95 | [24] |
| 22 | Ru/Nb_2_O_5_ | 90 | 41 | 6 | 98 | [25] |
| 23 | Ru/ZrO_2_ | 85 | 20 | 12 | 90 | [26] |
| 24 | Ni/Al_2_O_3_ | 80 | 10 | 20 | 99 | [27] |
| 25 | Rh/Al_2_O_3_ | 80 | >20 | 5 | 77 | [28] |
| 26 | Pt nanowire | 80 | >1 | 24 | 2 | [29] |
| 27 | NiAl-10 | 80 | 20 | 1 | 98 | [30] |
| 28 | Ru/BNC | 80 | 20 | 16 | 99 | [31] |
| 29 | Ru/TiP-100 | 30 | 10 | 15 | 96 | [32] |
| 30 | HAP-Pd | 25 | 1 | 2 | 0 | [33] |
| 31 | Rh/MFM-300(Cr) | 25 | 1 | 4 | 95 | This work |

3.2 Reductive amination over various catalysts

| **Table S2**. Products from reductive amination of cyclohexanone with various catalysts | | | | | | | | |
| --- | --- | --- | --- | --- | --- | --- | --- | --- |
|  | | | | | | | | |
| **Entry** | **Catalyst** | **Conversion (%)** | **Yield (%)** | | | | | |
|  |  |  | **P** | **SA** | **SB** | **IA** | **IB** | **IC** |
| 1 | null | 48 | 0 | 0 | 0 | 13 | 26 | <1 |
| 2 | Rh/MFM-300(Cr) | 100 | 98 | 0 | 2 | 0 | 0 | 0 |
| 3 | Pd/MFM-300(Cr) | 37 | 0 | 0 | 0 | 4 | 20 | 9 |
| 4 | Ru/MFM-300(Cr) | 41 | 0 | 0 | 0 | 6 | 23 | 1 |
| 5 | Pt/MFM-300(Cr) | 38 | 0 | 0 | 0 | 4 | 17 | 10 |
| 6 | Rh NPs (2.4 nm) | 47 | 0 | 0 | 0 | 1 | 5 | 37 |
| 7 | MFM-300(Cr) | 61 | 0 | 0 | 0 | 19 | 32 | <1 |
| 8 | Rh NPs+MFM-300(Cr) | 70 | 0 | 0 | 0 | 2 | 6 | 60 |
| 9 | Rh/Al_2_O_3_ | 77 | 0 | 0 | 0 | 1 | 3 | 74 |
| 10 | Rh/ZSM-5 | 75 | 1 | 0 | 0 | <1 | 1 | 68 |
| 11 | Rh/C | 25 | 0 | 0 | 0 | 1 | 14 | <1 |
| 12 | Rh/MIL-101(Cr) | 100 | 97 | 0 | 2 | 0 | 0 | <1 |
| 13 | Rh/Cr(OH)_3_ | 78 | 8 | 0 | 0 | 0 | <1 | 67 |
| 14 | Rh/Cr_2_O_3_ | 80 | 23 | 0 | 0 | 0 | <1 | 56 |
| 15 | Rh_2_O_3_ | 73 | 0 | 0 | 0 | 30 | 36 | <1 |
| 16 | Rh/MFM-300(Cr)-H_2_ | 69 | 0 | 0 | 0 | 25 | 28 | 9 |
| 17 | Rh/MFM-300(Cr)-DSM | 68 | 0 | 0 | 0 | 24 | 29 | 13 |
| Reaction conditions: 3.5 mol/L NH_3_/MeOH solution (4 mL), cyclohexanone (1.0 mmol), catalyst 0.2 mol%, 1 bar H_2_, 25 ^o^C, 3 h. | | | | | | | | |

**Catalytic system optimization.** MFM-300(Cr), [Cr_2_(OH)_2_(L)] (L^4-^ = biphenyl-3,3’,5,5’-tetracarboxylate), is comprised of chains of [Cr(OH)_2_O_4_]_∞_ bridged by tetracarboxylate ligands. This structure affords channels bounded by Cr-O(H)-Cr groups and phenyl rings in a ‘wine rack’ array, and showing exceptional stability to NH_3_. Rh/MFM-300(Cr) (Rh loading of 2 wt%) was prepared *via* an impregnation method with RhCl_3_ followed by reduction with NaBH_4_ (see Methods for details). For comparison, M/MFM-300(Cr) (M = Pt, Pd, Ru) and Rh/support [support = Al_2_O_3_, ZSM-5, activated carbon, MIL-101(Cr), Cr(OH)_3_, Cr_2_O_3_] as well as bare Rh nanoparticles and Rh_2_O_3_ have also been synthesised and investigated for catalytic activity. Cyclohexanone was chosen as the model compound to evaluate the performance of these catalysts for reductive amination with NH_3_ (3.5 mol L^-1^ in MeOH) and H_2_ (1 bar) at 25 ^o^C for 3 h (Table S2). The possible products include primary amine (cyclohexanamine, **P**), secondary amine (dicyclohexylamine, **SA**) and alcohol (cyclohexanol, **SB**) and possible intermediates include imine (cyclohexanimine, **IA**), Schiff-base [N-(cyclohex-1-en-1-yl)cyclohexanimine, **IB,** and N-cyclohexylcyclohexanimine, **IC**].^[34]^ In the absence of catalyst, cyclohexanone can be partially (48%) converted to the intermediates **IA** and **IB** by reacting with NH_3_ (entry 1, Table S2). Importantly, in the presence of Rh/MFM-300(Cr) full conversion and high selectivity (98%) to **P** is observed with only a trace amount of **SB** (2%) formed as bi-product (entry 2, Table S2). By contrast, M/MFM-300(Cr) (M = Pt, Pd and Ru) show only moderate conversion (37-41%) and no product **P** (entries 3-5, Table S2). Rh nanoparticles (2.4 nm) alone yield only intermediate **IC** of 37% (entry 6, Table S2). Likewise, bare MFM-300(Cr) also gives intermediates **IA** and **IB** (19% and 32%, respectively; entry 7, Table S2). Similarly, a powdered mixture of Rh nanoparticles (2.4 nm) and MFM-300(Cr) gives intermediates **IA**, **IB** and **IC** (2%, 6% and 60%, respectively; entry 8, Table S2), indicating the presence of the synergetic effects Rh nanoparticles supported by MFM-300(Cr). Activated carbon, Al_2_O_3_, and ZSM-5 supported Rh also show low activities (entries 9-11, Table S2). Interestingly, the catalysts based upon other Cr-based supports [*i.e.*, Rh/MIL-101(Cr), Rh/Cr_2_O_3_ and Rh/Cr(OH)_3_] showed improved yields of the primary amine **P** (97%, 8% and 23%, respectively; entries 12-14, Table S2). Notably, Rh/MIL-101(Cr) shows a similar catalytic performance to Rh/MFM-300(Cr).

The method of reduction also has a great influence on the activity of Rh/MFM-300(Cr). While Rh/MFM-300(Cr) reduced by NaBH_4_ showed a high yield of **P** (98%), reduction using H_2_ gave a completely inactive species under the same conditions (entry 16, Table S2). The double solvent method (DSM) has been demonstrated as an effective strategy to encapsulate nanoparticles into the pores of porous materials.^[35]^ Rh/MFM-300(Cr) prepared by this method is completely inactive (entry 17, Table S2), perhaps suggesting that the reaction is taking place mainly on the external surface of the Rh-doped material with the MOF acting as a surface support for active Rh particles (see below).

Carrying out the reaction with H_2_ diluted to 30% in Ar at 1 bar affords the primary amine up to 98% yield but with an elongated reaction time of 12 h (Table S3). The concentration of NH_3_ solution also affects the performance, but mainly on the activity rather than selectivity (Table S3). Thus, the selectivity to primary amine of Rh/MFM-300(Cr) is independent to the pressure of H_2_ and NH_3_, and therefore precise control of H_2_ and NH_3_ concentrations is not necessarily required to optimise selectivity. We envisage that the raw mixtures of H_2_, N_2_ and NH_3_ can thus be used to drive this reaction without the need for the challenging pre-separations of these substrates. The catalyst Rh/MFM-300(Cr) can be recycled 10 times with only minor reduction in the catalytic activity (92% after 10 cycles, Figure S2). These results clearly demonstrate the exceptional catalytic performance of Rh/MFM-300(Cr) for reductive amination with H_2_ and NH_3_ under ambient conditions.

**Role of Rh(0) and Rh(III) species**. To clarify the individual contributions of Rh(0) and Rh(III) species, we further studied catalysts with distinct Rh oxidation states and configurations. Under standard conditions (3.5 M NH_3_/MeOH, cyclohexanone 1.0 mmol, 0.2 mol% catalyst, H_2_ 1 bar, 25 °C, 3 h), Rh/MFM-300(Cr) achieved complete conversion and 98% yield of the desired primary amine (Entry 2), markedly outperforming both other noble metals and Rh catalysts supported on conventional materials (Entries 3–5, 9–11). In contrast, isolated Rh nanoparticles (NPs) and a physical mixture of Rh NPs with MFM-300(Cr) (Table S2, Entries 6 and 8) afforded only 47% and 70% conversion, respectively, without detectable cyclohexylamine formation. These results indicate that the synergistic integration of Rh species with the MOF support is crucial for amination activity. Notably, the presence of MFM-300(Cr) increased the yield of the imine intermediate from 37% (Rh NPs alone) to 60%, suggesting that the framework stabilizes the imine intermediate. When Rh_2_O_3_ was used as the catalyst, no cyclohexylamine or other hydrogenation products were detected, confirming that Rh(III) alone is inactive for H_2_ activation. However, ~30% yield of intermediate IA and 36% yield of intermediate IB were observed, which typically formed via condensation of cyclohexanone and NH₃ catalyzed by Lewis acid sites. This observation supports the role of Rh(III) as a Lewis acid center facilitating imine (Schiff base) formation. Conversely, the Rh/MFM-300(Cr)-H₂ catalyst, prepared by high-temperature H₂ reduction and thus enriched in Rh(0) sites, exhibited a Rh(0)/Rh(III) ratio higher than the optimal catalyst but delivered only a 9% yield of cyclohexylamine. This finding indicates that Rh(III) acts as a Lewis acid but also modulates the electronic state of Rh(0), and an appropriate Rh(III)/Rh(0) ratio is essential to balance hydrogenation activity and substrate activation, consistent with XPS analysis.

Collectively, these results, together with the XPS and INS analyses, reveal that Rh(III) functions as a Lewis acid site to activate the carbonyl group of aldehydes/ketones and promote imine formation, whereas Rh(0) acts as the hydrogenation center that converts imine or gem-diamine intermediates into amines. The synergistic interplay between Rh(III) and Rh(0) finely tunes the electronic environment of Rh(0), in concert with MFM-300(Cr), optimizing substrate adsorption, intermediate stabilization, and product desorption, thereby achieving both high activity and selectivity.

3.3 Effect of NH_3_ and H_2_ pressure

**Table S3. Effect of NH_3_ concentration and H_2_ partial pressure on the reductive amination of cyclohexanone**

|  | H_2_ Source (1 bar) | NH_3_ concentration (mol/L) | Time (h) | Yield of Primary amine | Yield of Secondary amine |
| --- | --- | --- | --- | --- | --- |
| 1 | Pure H_2_ | 3.5 | 3 | 98% | 0 |
| 2 | 50% H_2_/Ar | 3.5 | 12 | 98% | 0 |
| 3 | 30% H_2_/Ar | 3.5 | 12 | 98% | 0 |
| 4 | 5% H_2_/Ar | 3.5 | 12 | <1% | 0 |
| 5 | Pure H_2_ | 7 | 3 | 98% | 0 |
| 6 | Pure H_2_ | 1.7 | 4 | 98% | 0 |
| 7 | Pure H_2_ | 0.9 | 12 | 98% | 0 |
| 8 | Pure H_2_ | 0.4 | 12 | 23% | 0 |

3.4 Assignment of vibrational modes in INS

**Table S4. Assignment of vibration modes of BPDI**

| Simulated | experimental | Vibrational mode |
| --- | --- | --- |
| 205 | 205 | C-C-C twisting |
| 279 | 258 | NH_2_ torsion |
| 297 | 280 | C-C-C twisting |
| 758 | 744 | N-H rocking |
| 791 | 778 | NH_2_ wagging |
| 914 | 883 | N-H wagging of NH_2_ and C-H rocking of CH_2_ |
| 932 | 926 | N-C-N symmetrical stretching |
| 1070 | 1090 | C-N stretching |
| 1130 | 1120 | N-C-N asymmetrical stretching |
| 1185 | 1178 | N_16_-H twisting coupled with C_9_-H rocking |
| 1239 | 1260 | C-N_16_ stretching coupled with C_7_-H, N16-H twisting and C-H, N-H rocking |
| 1334 | 1327 | CH_2_ wagging couple with C-H rocking |
| 1373 | 1365 | C_9_-H rocking coupled with NH_2_ twisting |
| 1440 | 1433 | C-H scissoring |

**Table S5. Assignment of vibration modes of BPI**

| Simulated | experimental | Vibrational mode |
| --- | --- | --- |
| 299 | 292 | C-C-C wagging |
| 497 | 487 | C-C-C-C wagging |
| 616 | 616 | Ring deformation |
| 665 | 643 | C-H wagging in benzene ring |
| 738 | 694 | C-H wagging in benzene ring |
| 772 | 755 | C-H wagging in benzene ring |
| 849 | 845 | C-H wagging in benzene ring |
| 906 | 911 | C-H wagging in benzene ring |
| 990 | 980 | C-C bending and C-H rocking in benzene ring and C9-H rocking |
| 1029 | 1026 | C-N stretching |
| 1084 | 1075 | C-H rocking |
| 1168 | 1165 | C-H rocking of benzene ring |
| 1214 | 1208 | C7-H twisting |
| 1330 | 1333 | C7-H wagging |
| 1382 | 1376 | C9-H rocking |
| 1450 | 1445 | C-H scissoring |
| 1494 | 1494 | C-H in plane rocking of benzene ring |

**Table S6. Assignment of vibration modes of BA**

| Simulated | experimental | Vibrational mode |
| --- | --- | --- |
| 270 | 275 | NH_2_ torsion |
| 405 | 404 | C-C-C twisting in benzene ring |
| 456 | 465 | C out of plane wagging in benzene ring |
| 567 | 575 | ring deformation |
| 695 | 699 | C-C wagging in benzene ring |
| 732 | 738 | C-H wagging in benzene ring |
| 862 | 849 | NH_2_ wagging coupled with CH_2_ rocking |
| 915 | 902 | (C-H twisting in benzene ring) |
| 989 | 979 | (C-H twisting in benzene ring) |
| 1061 | 1055 | C-N stretching |
| 1074 | 1093 | C-H rocking in benzene ring |
| 1154 | 1167 | NH_2_ twisting coupled with CH_2_ twisting |
| 1398 | 1388 | C-H wagging coupled with NH_2_ twisting |
| 1462 | 1460 | C-H scissoring |

4. References

[1] G. W. T. M. J. Frisch, H. B. Schlegel, G. E. Scuseria, M. A. Robb, J. R. Cheeseman, G. Scalmani, V. Barone, G. A. Petersson, H. Nakatsuji, X. Li, M. Caricato, A. V. Marenich, J. Bloino, B. G. Janesko, R. Gomperts, B. Mennucci, H. P. Hratchian, J. V. Ortiz, A. F. Izmaylov, J. L. Sonnenberg, D. Williams-Young, F. Ding, F. Lipparini, F. Egidi, J. Goings, B. Peng, A. Petrone, T. Henderson, D. Ranasinghe, V. G. Zakrzewski, J. Gao, N. Rega, G. Zheng, W. Liang, M. Hada, M. Ehara, K. Toyota, R. Fukuda, J. Hasegawa, M. Ishida, T. Nakajima, Y. Honda, O. Kitao, H. Nakai, T. Vreven, K. Throssell, J. A. Montgomery, Jr., J. E. Peralta, F. Ogliaro, M. J. Bearpark, J. J. Heyd, E. N. Brothers, K. N. Kudin, V. N. Staroverov, T. A. Keith, R. Kobayashi, J. Normand, K. Raghavachari, A. P. Rendell, J. C. Burant, S. S. Iyengar, J. Tomasi, M. Cossi, J. M. Millam, M. Klene, C. Adamo, R. Cammi, J. W. Ochterski, R. L. Martin, K. Morokuma, O. Farkas, J. B. Foresman, D. J. Fox, Gaussian, Inc., Wallingford CT, Gaussian 16, Revision A.03, 2016. **2016**.

[2] A. J. Ramirez-Cuesta, *Comput. Phys. Commun*. 2004, *157*, 226-238.

[3] L. F. Lin, A. M. Sheveleva, I. da Silva, C. M. A. Parlett, Z. M. Tang, Y. M. Liu, M. T. Fan, X. Han, J. H. Carter, F. Tuna, E. J. L. McInnes, Y. Q. Cheng, L. L. Daemen, S. Rudic, A. J. Ramirez-Cuesta, C. C. Tang, S. H. Yang, *Nat. Mater*. 2020, *19*, 86-93.

[4] T. Tsuda, H. Ishikawa, M. Sheng, M. Hirayama, S. Suganuma, R. Osuga, K. Nakajima, J. N. Kondo, S. Yamaguchi, T. Mizugaki, T. Mitsudome, *J. Am. Chem. Soc*. 2025, *147*, 14326-14335.

[5] Y. Nakamura, K. Kon, A. S. Touchy, K. Shimizu, W. Ueda, *ChemCatChem*, 2015, *7*, 921-924.

[6] T. Gross, A. M. Seayad, M. Ahmad, M. Beller, *Org. Lett*. 2002, *4*, 2055-2058.

[7] C. Bäumler, C. Bauer, R. Kempe, *ChemSusChem*, 2020, *13*, 3110-3114.

[8] T. Senthamarai, K. Murugesan, J. Schneidewind, N. V. Kalevaru, W. Baumann, H. Neumann, P. C. J. Kamer, M. Beller, R. V. Jagadeesh, *Nat. Commun*. 2018, *9*, 4123.

[9] J. Gallardo-Donaire, M. Ernst, O. Trapp, T. Schaub, *Adv. Synth. Catal*. 2016, *358*, 358-363.

[10] K. Murugesan, M. Beller, R. V. Jagadeesh, *Angew. Chem. Int. Ed*. 2019, *58*, 5064-5068.

[11] R. V. Jagadeesh, K. Murugesan, A. S. Alshammari, H. Neumann, M. M. Pohl, J. Radnik, M. Beller, *Science*, 2017, *358*, 326-332.

[12] K. Murugesan, V. G. Chandrashekhar, T. Senthamarai, R. V. Jagadeesh, M. Beller, *Nat. Protoc*. 2020, *15*, 1313-1337.

[13] X. Zhuang, X. Wei, X. Hu, Q. Zhang, X. Zhang, L. Chen, J. Liu, L. Ma, *Green Chem*. 2023, *25*, 7109-7125.

[14] M. Sheng, S. Fujita, S. Yamaguchi, J. Yamasaki, K. Nakajima, S. Yamazoe, T. Mizugaki, T. Mitsudome, *JACS Au*, 2021, *1*, 501-507.

[15] K. Murugesan, Z. H. Wei, V. G. Chandrashekhar, H. Neumann, A. Spannenberg, H. J. Jiao, M. Beller, R. V. Jagadeesh, *Nat. Commun*. 2019, *10*, 5443

[16] H. F. Qi, J. Yang, F. Liu, L. L. Zhang, J. Y. Yang, X. Y. Liu, L. Li, Y. Su, Y. F. Liu, R. Hao, A. Q. Wang, T. Zhang, *Nat. Commun*. 2021, *12*, 3295

[17] S. L. Lu, P. Y. Xu, X. Q. Cao, H. W. Gu, *RSC Adv*. 2018, *8*, 8755-8760.

[18] S. Nishimura, K. Mizuhori, K. Ebitani, *Res. Chem. Intermed*. 2016, *42*, 19-30.

[19] R. Shang, H. Zhang, Y. Li, B. Gu, Q. Tang, F. Qiu, Q.-E. Cao, W. Fang, *ACS Catal*. 2025, *15*, 13322-13336.

[20] S. Gomez, J. A. Peters, J. C. van der Waal, W. Z. Zhou, T. Maschmeyer, *Catal. Lett*. 2002, *84*, 1-5.

[21] A. W. Heinen, J. A. Peters, H. van Bekkum, Eur. *J. Org. Chem*. 2000, *2000*, 2501-2506.

[22] K. Han, S. Song, Y. You, X. Fu, X. Li, *ACS Catal*. 2025, *15*, 14575-14587.

[23] G. Z. Xu, Z. H. Tu, X. B. Hu, X. M. Zhang, Y. T. Wu, *Chem. Eng. J*. 2024, *481*, 148704.

[24] D. Chandra, Y. Inoue, M. Sasase, M. Kitano, A. Bhaumik, K. Kamata, H. Hosono, M. Hara, *Chem. Sci*. 2018, *9*, 5949-5956.

[25] T. Komanoya, T. Kinemura, Y. Kita, K. Kamata, M. Hara, *J. Am. Chem. Soc*. 2017, *139*, 11493-11499.

[26] G. F. Liang, A. Q. Wang, L. Li, G. Xu, N. Yan, T. Zhang, *Angew. Chem. Int. Ed*. 2017, *56*, 3050-3054.

[27] G. Hahn, P. Kunnas, N. de Jonge, R. Kempe, *Nat. Catal*. 2019, *2*, 71-77.

[28] M. Chatterjee, T. Ishizaka, H. Kawanami, *Green Chem*. 2016, *18*, 487-496.

[29] F. Q. Qi, L. Hu, S. L. Lu, X. Q. Cao, H. W. Gu, *Chem. Commun*. 2012, *48*, 9631-9633.

[30] Z. Pan, Q. Zhang, W. Wang, L. Wang, G.-H. Wang, *ACS Sustain. Chem. Eng*. 2022, *10*, 3777-3786.

[31] H. T. Zou, J. Z. Chen, *Appl. Catal. B Environ. Energy*, 2022, *309*, 121262.

[32] C. Xie, J. L. Song, M. L. Hua, Y. Hu, X. Huang, H. R. Wu, G. Y. Yang, B. X. Han, *ACS Catal*. 2020, *10*, 7763-7772.

[33] Z. L. Yuan, P. Zhou, X. X. Liu, Y. X. Wang, B. Liu, X. Li, Z. H. Zhang, *Ind. Eng. Chem. Res*. 2017, *56*, 14766-14770.

[34] C. Xie, J. Song, M. Hua, Y. Hu, X. Huang, H. Wu, G. Yang, B. Han, *ACS Catal*. 2020, *10*, 7763-7772.

[35] A. Aijaz, A. Karkamkar, Y. J. Choi, N. Tsumori, E. Rönnebro, T. Autrey, H. Shioyama, Q. *J. Am. Chem. Soc*. 2012, *134*, 13926-13929.

5. NMR spectra of substrates


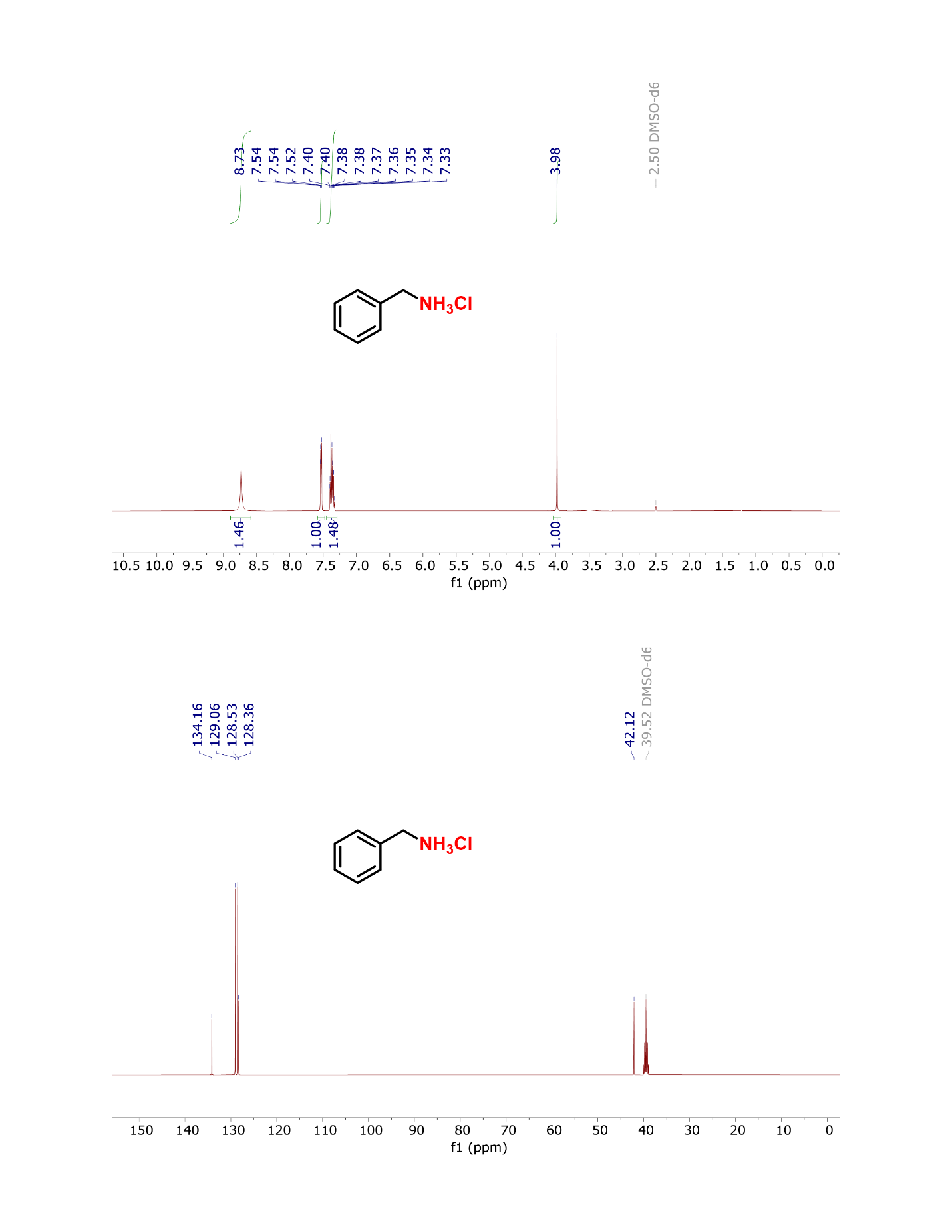


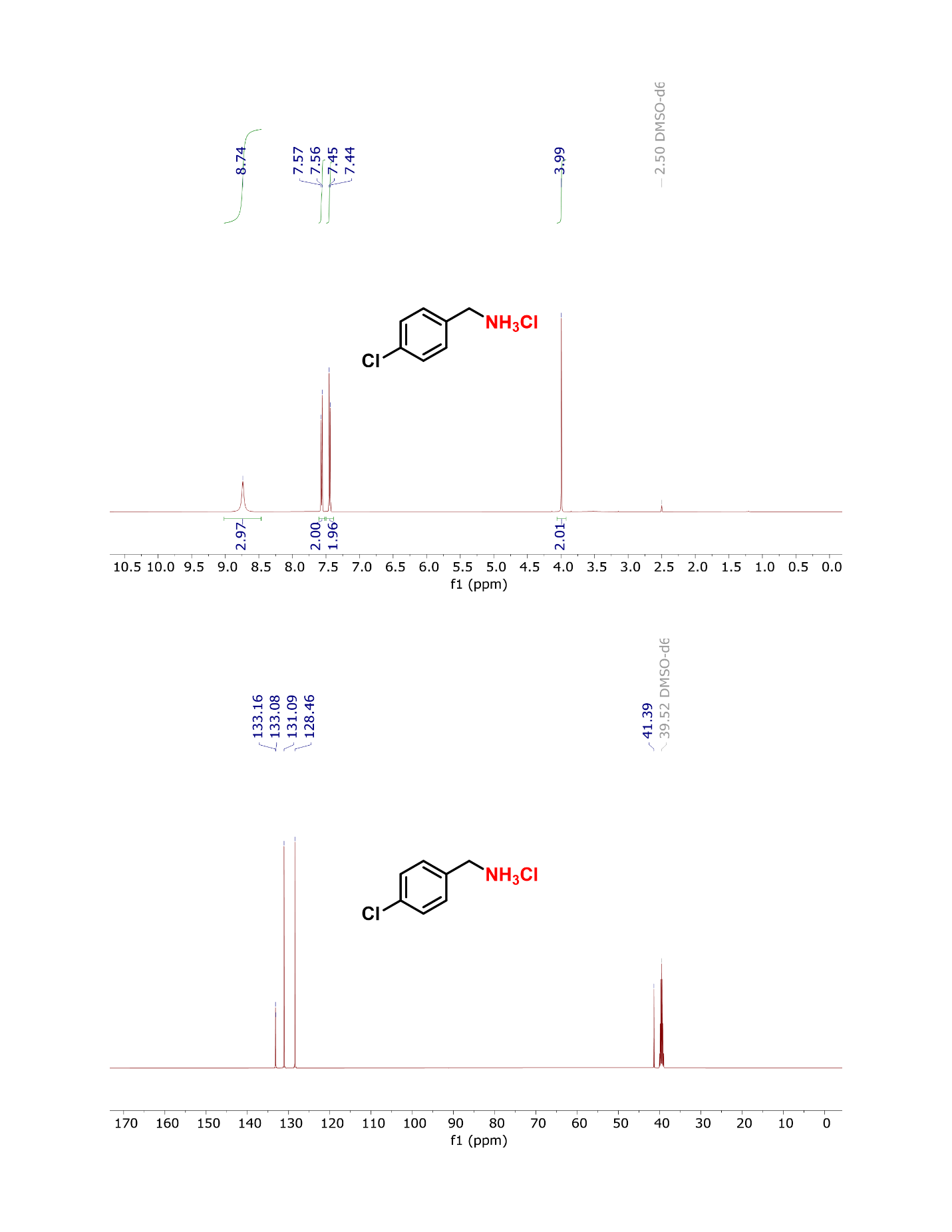


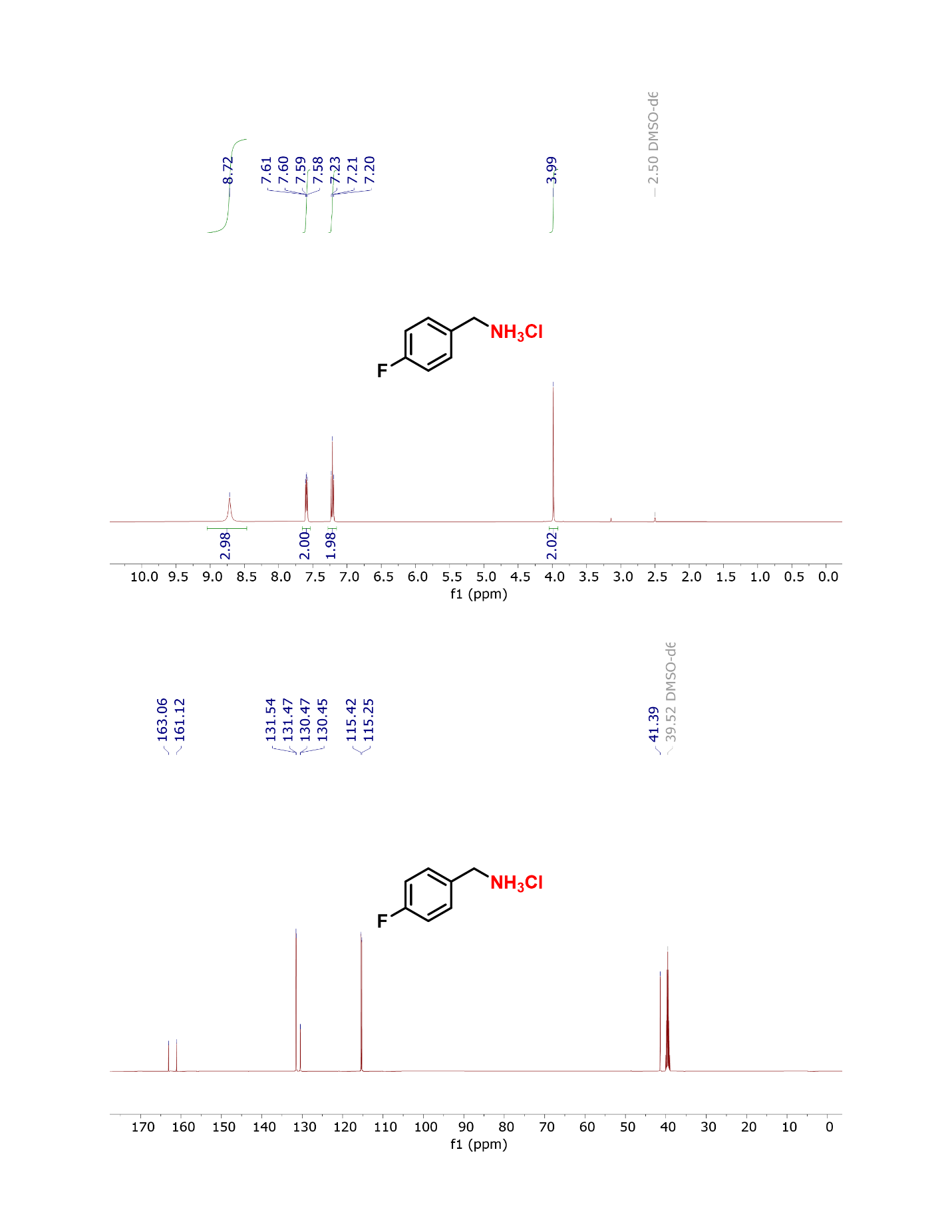


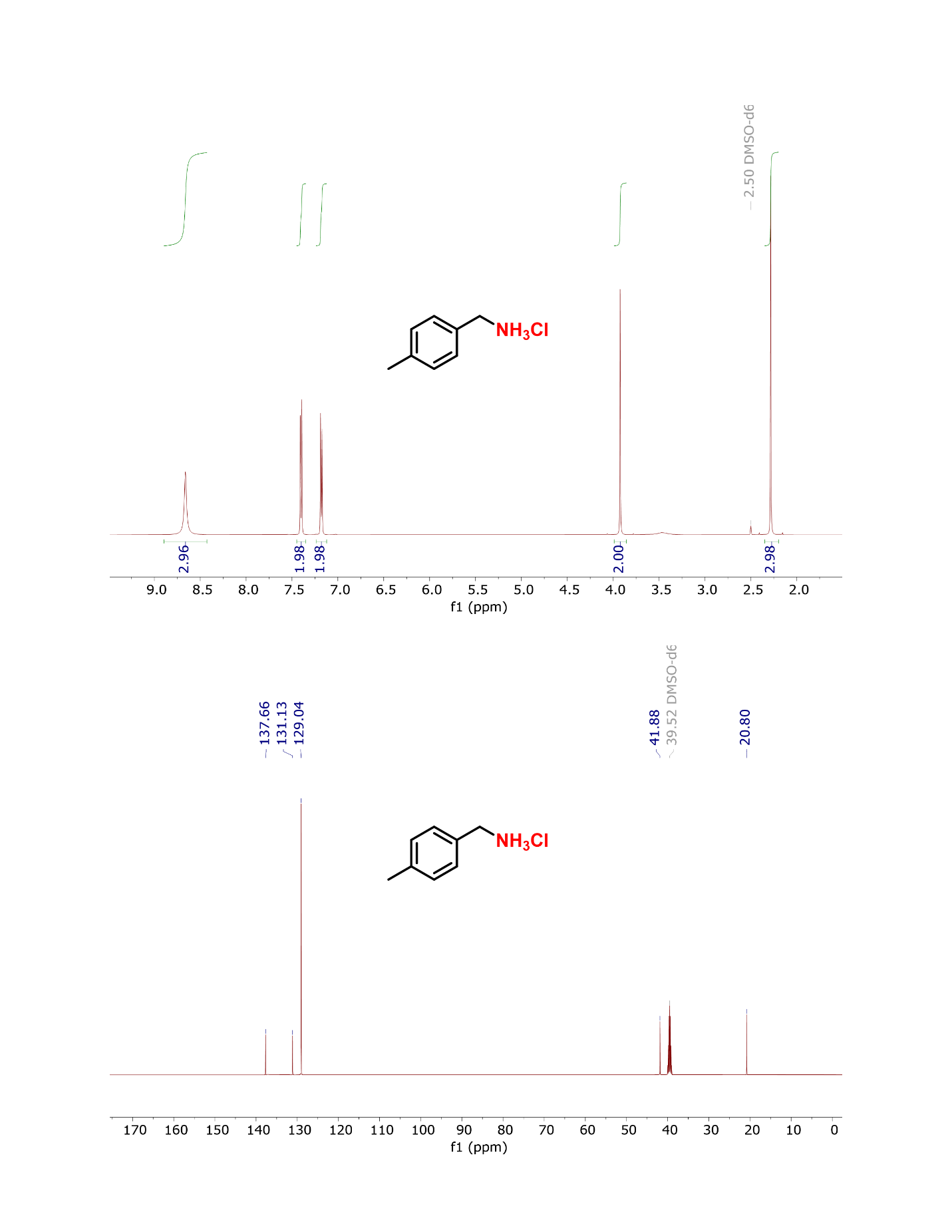


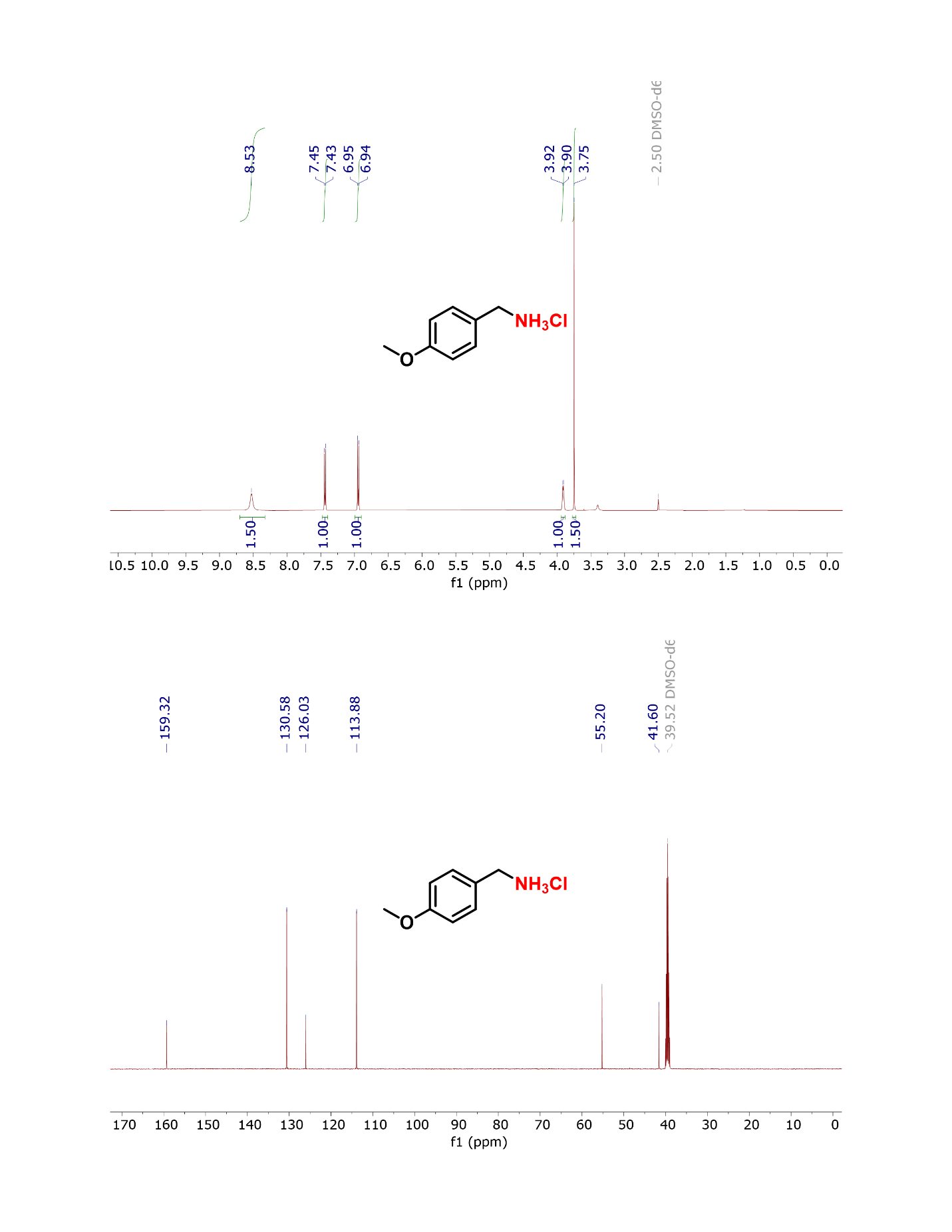


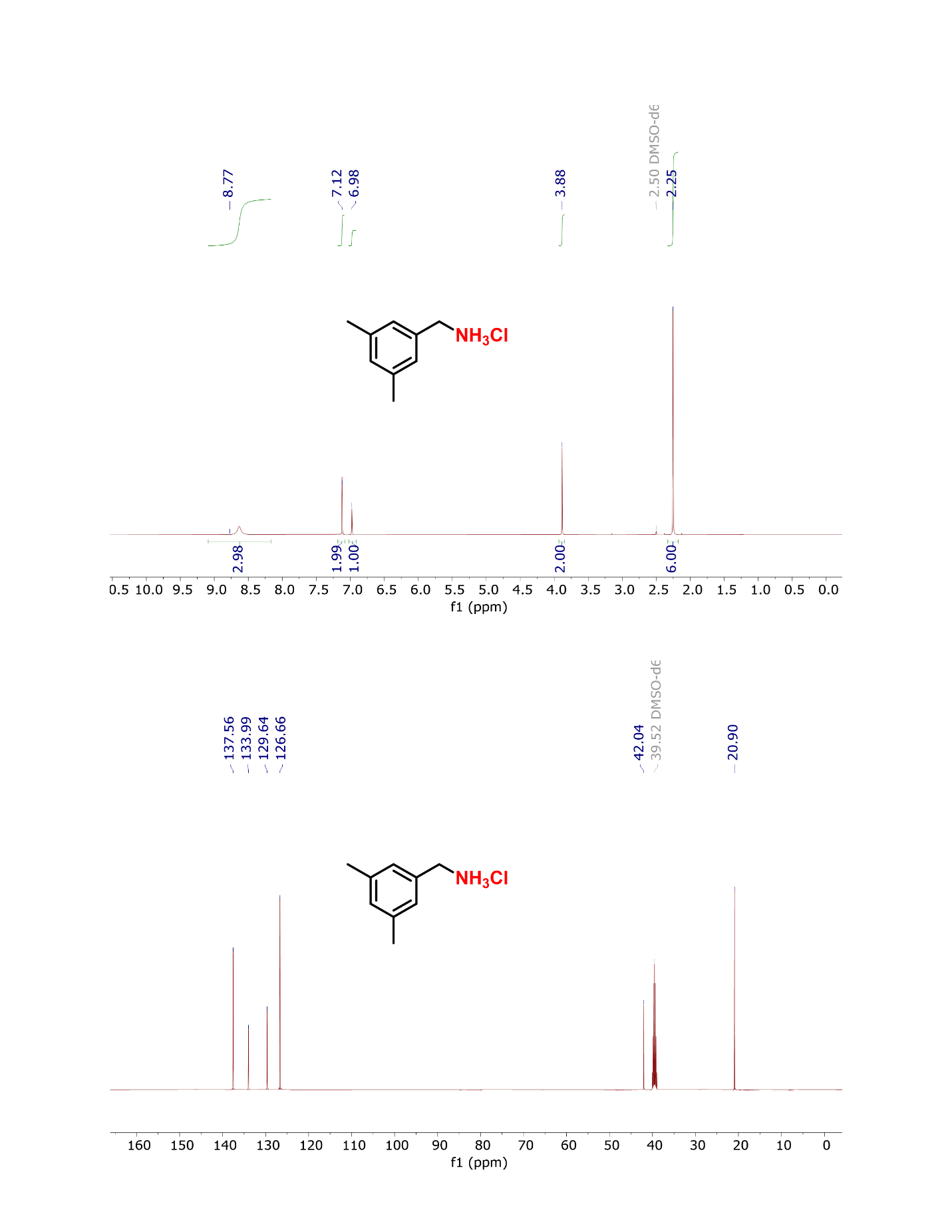


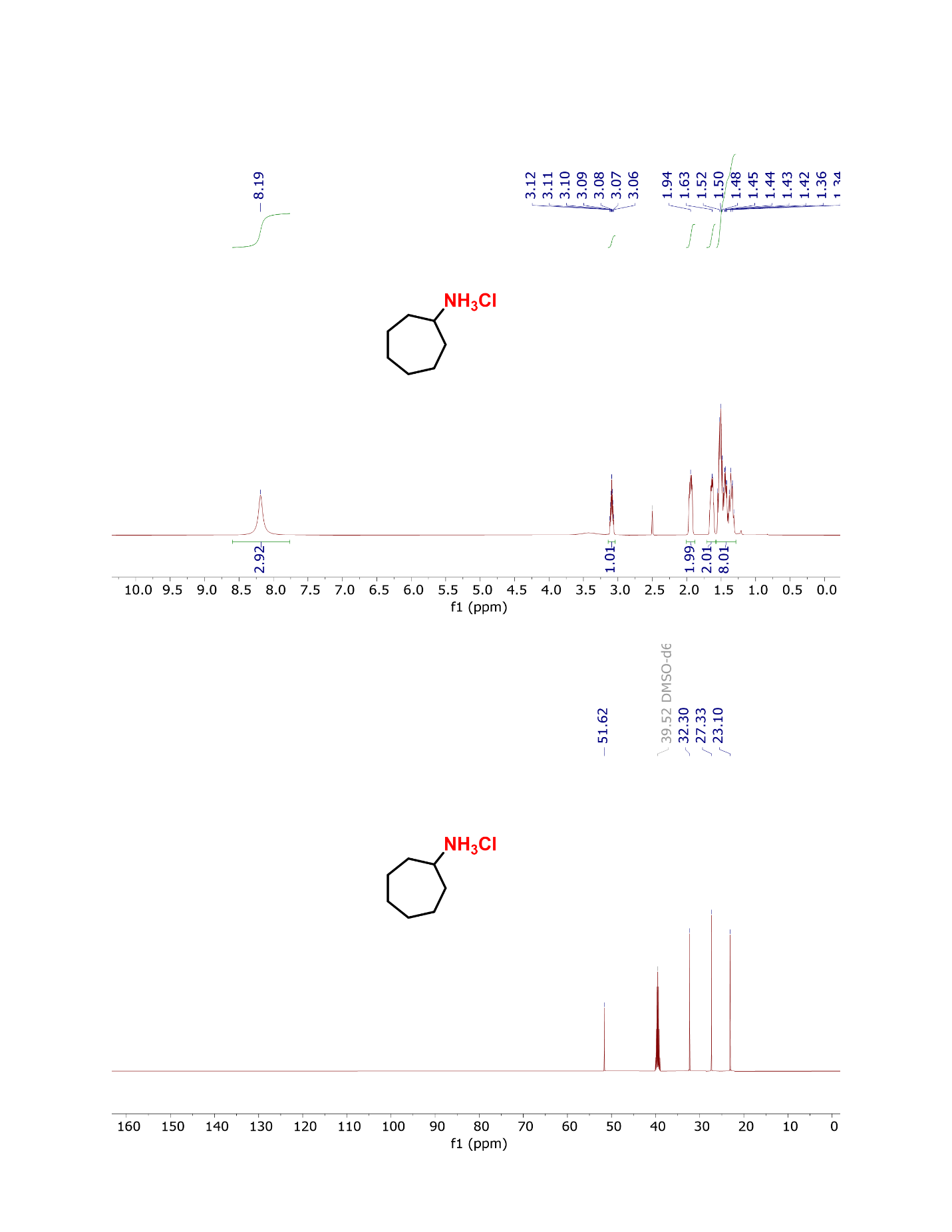


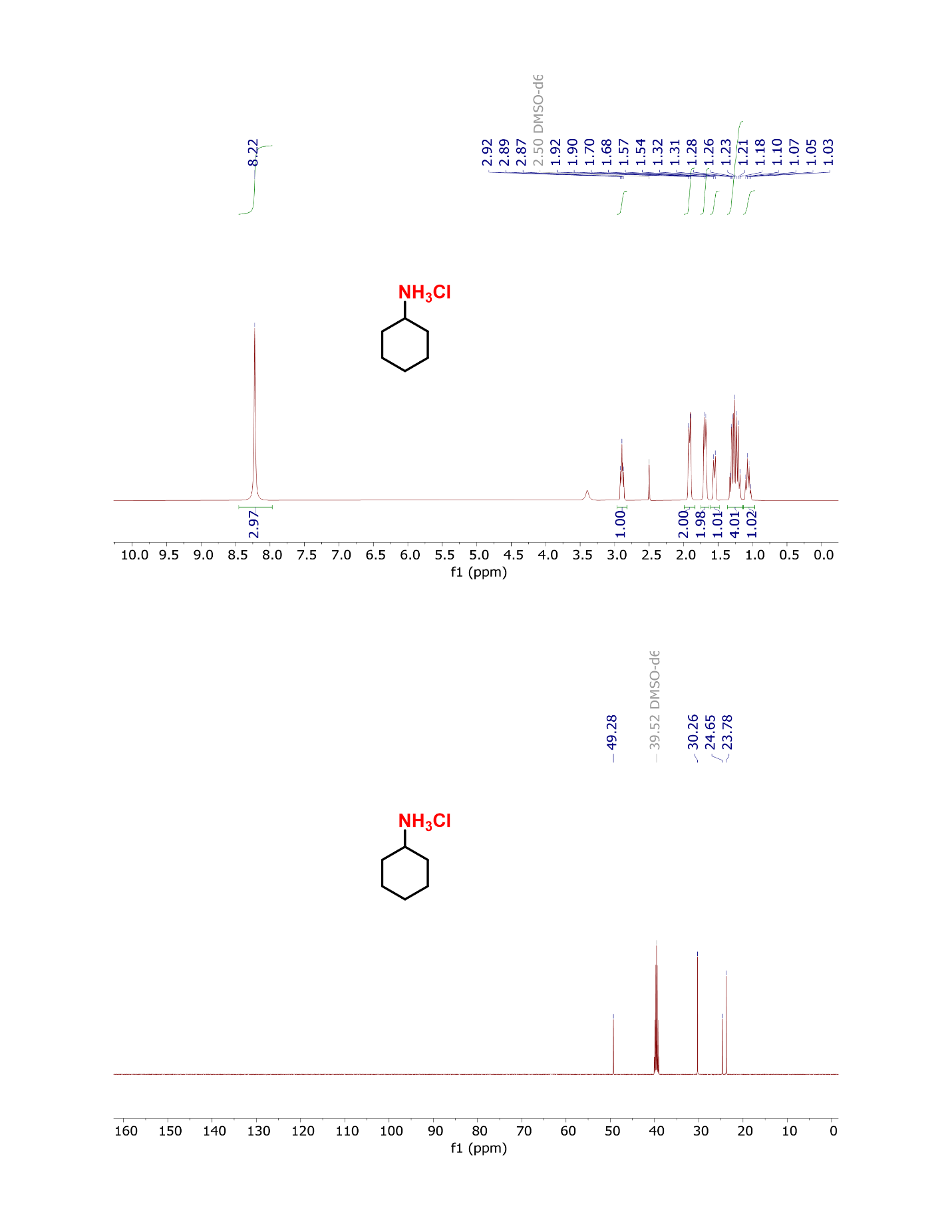


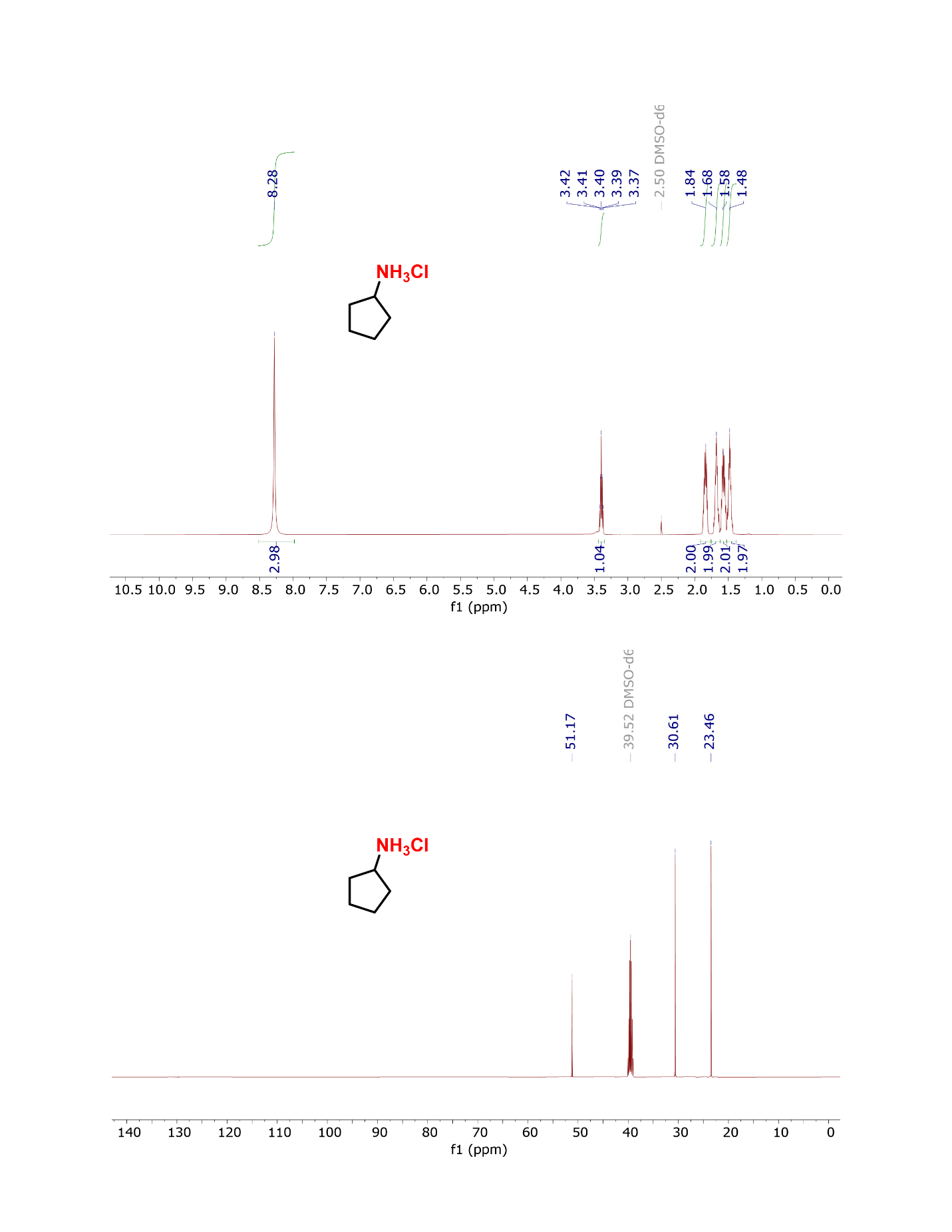


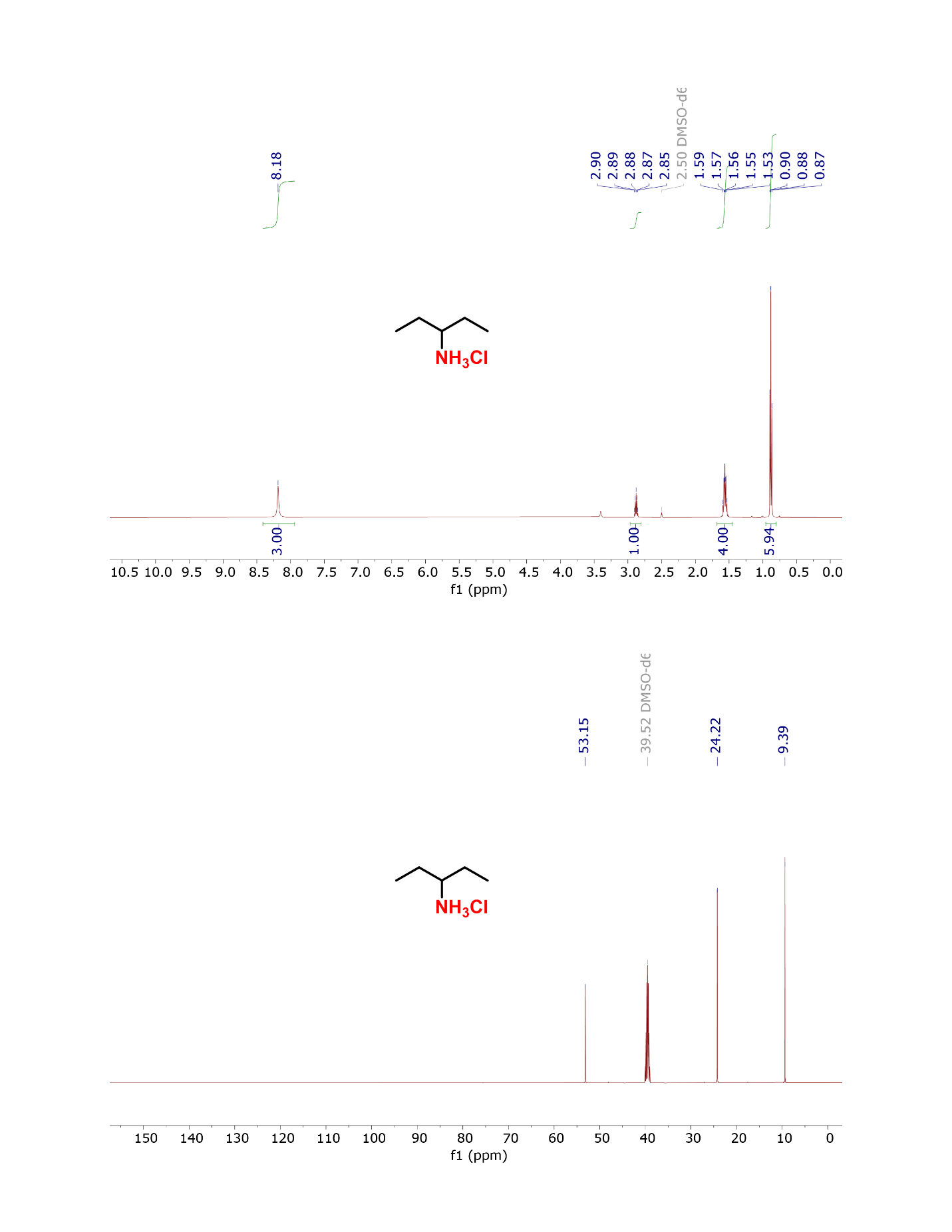


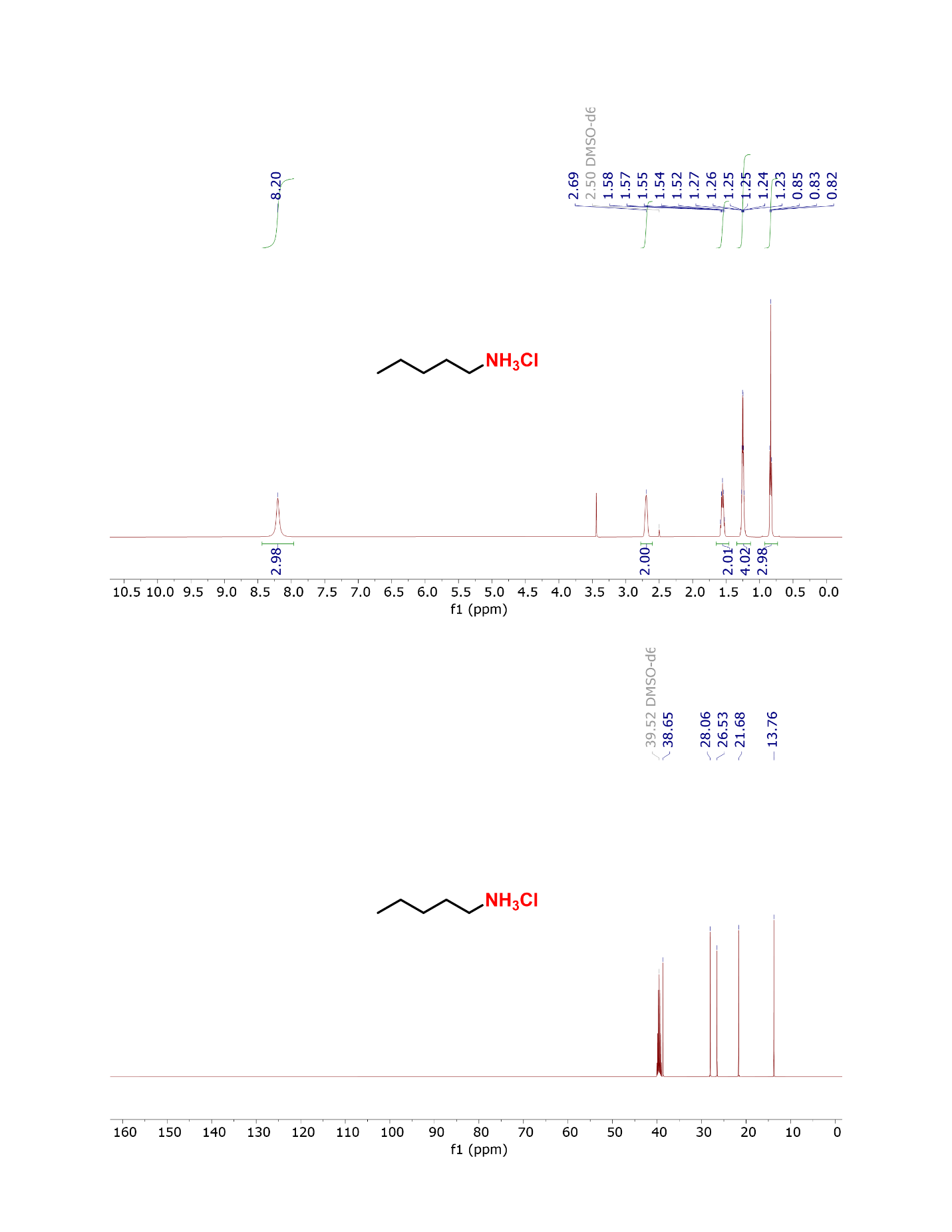


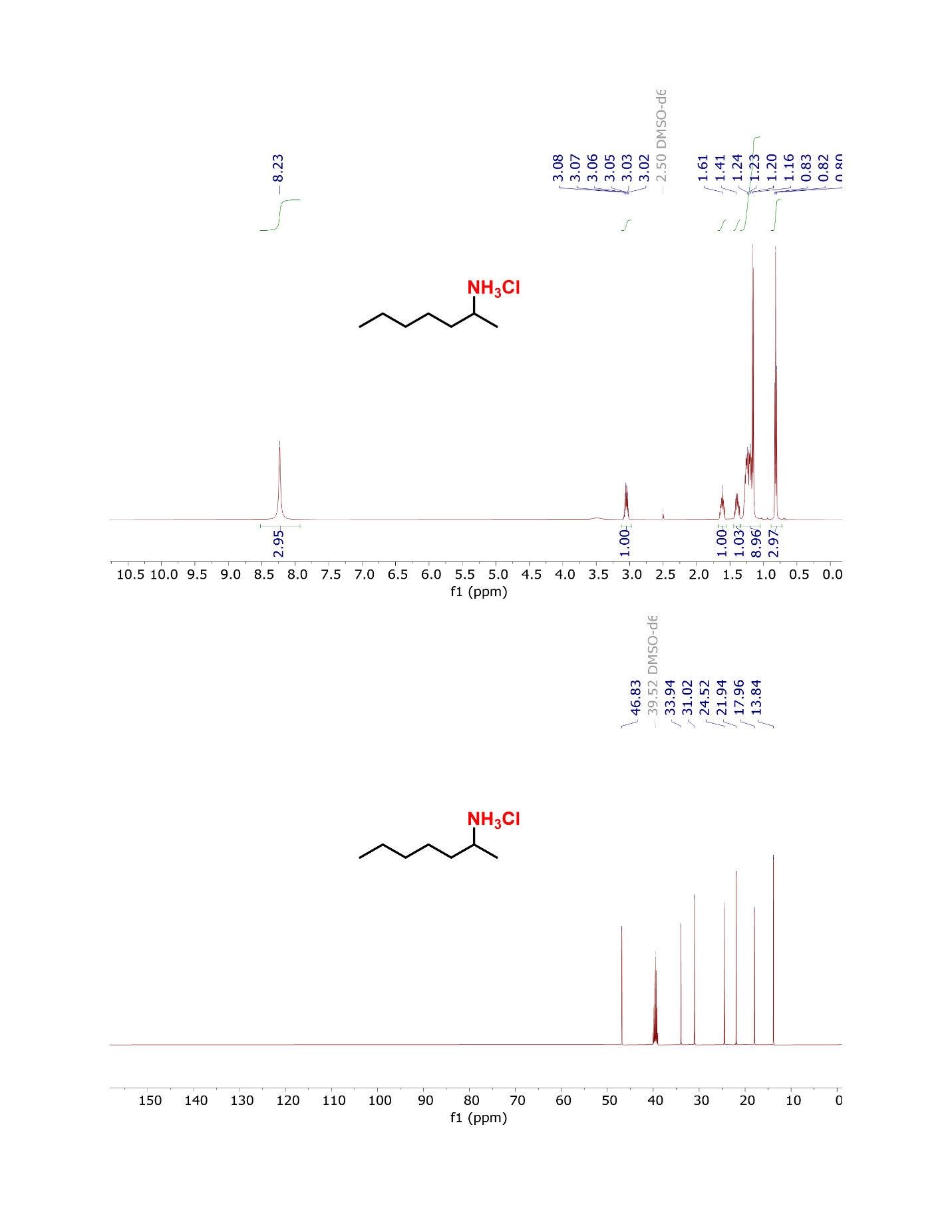


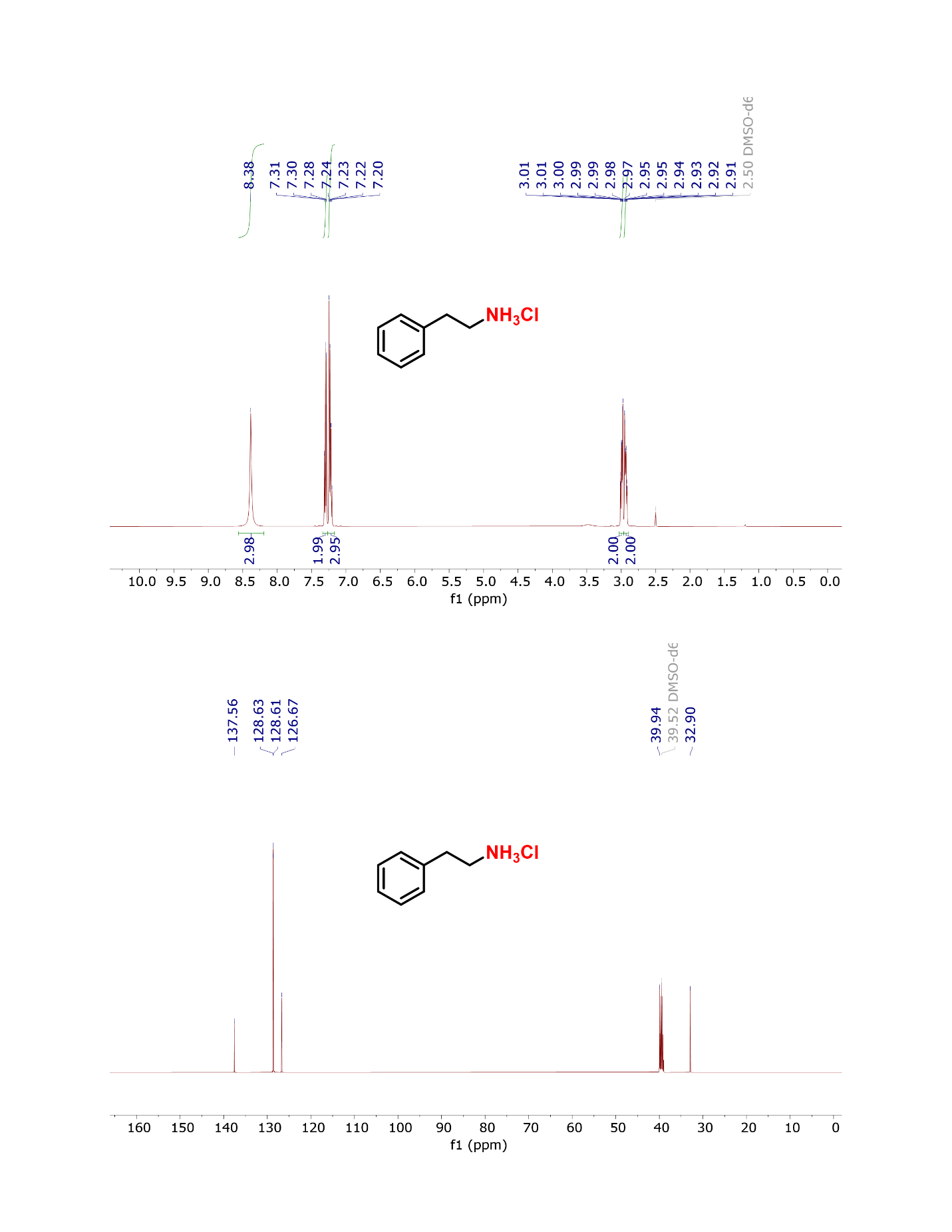


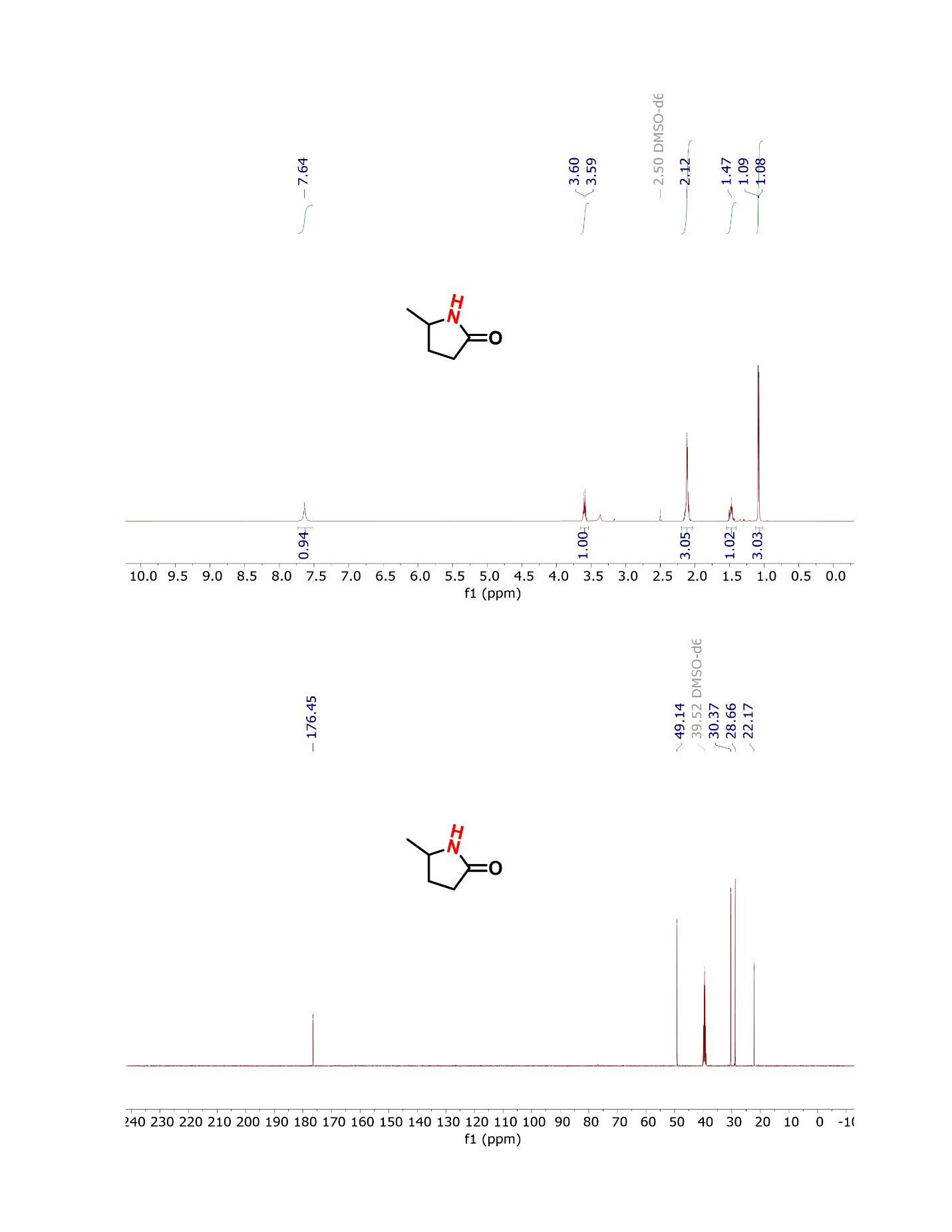


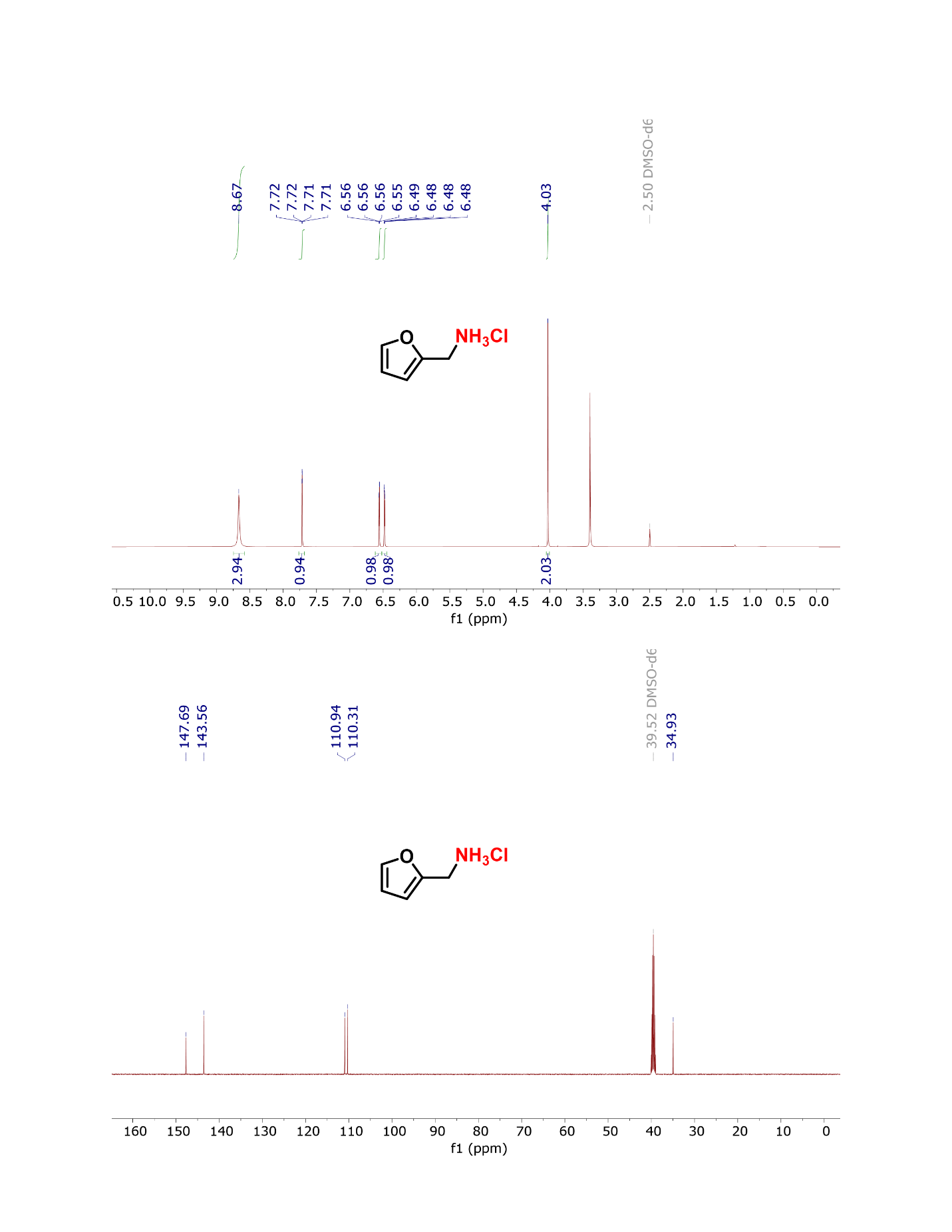


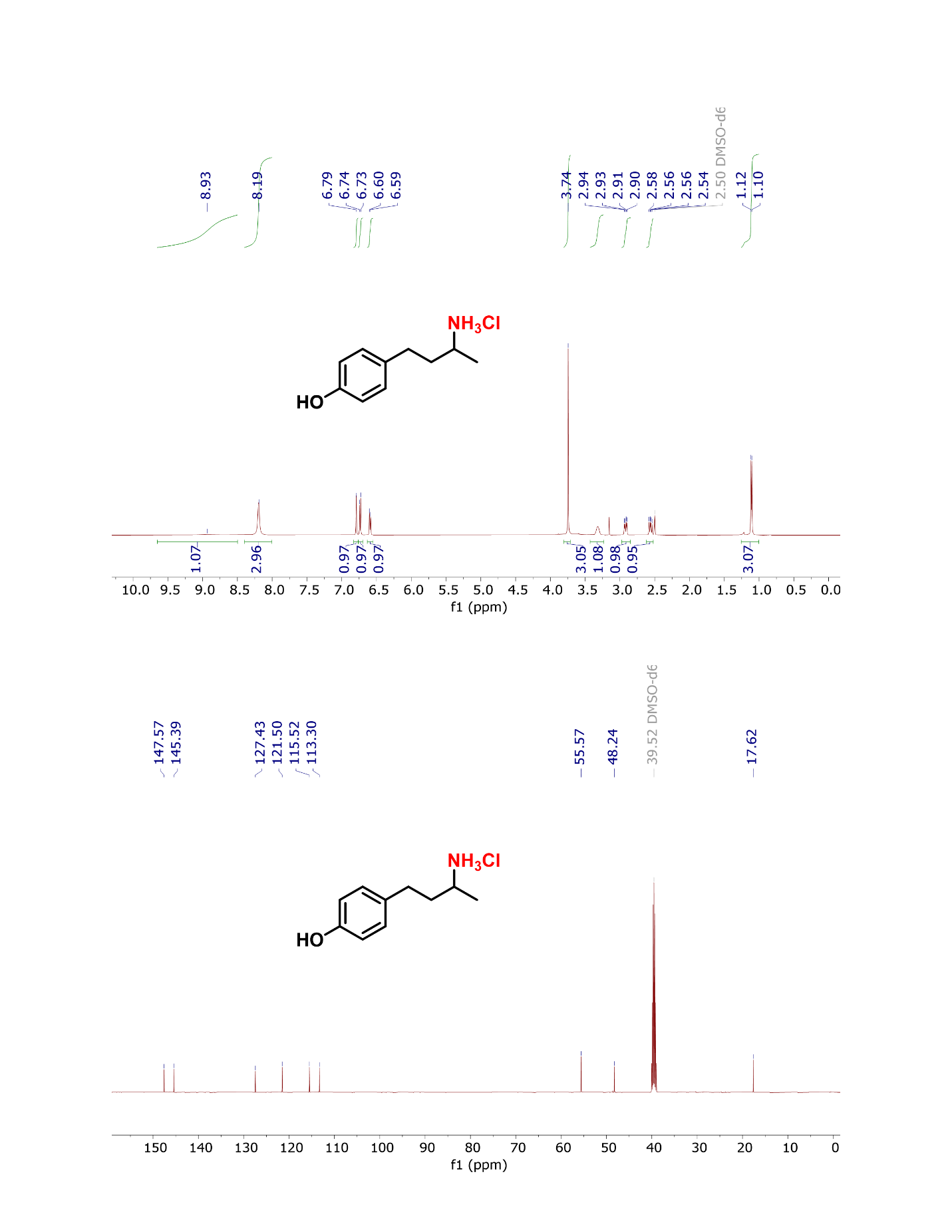


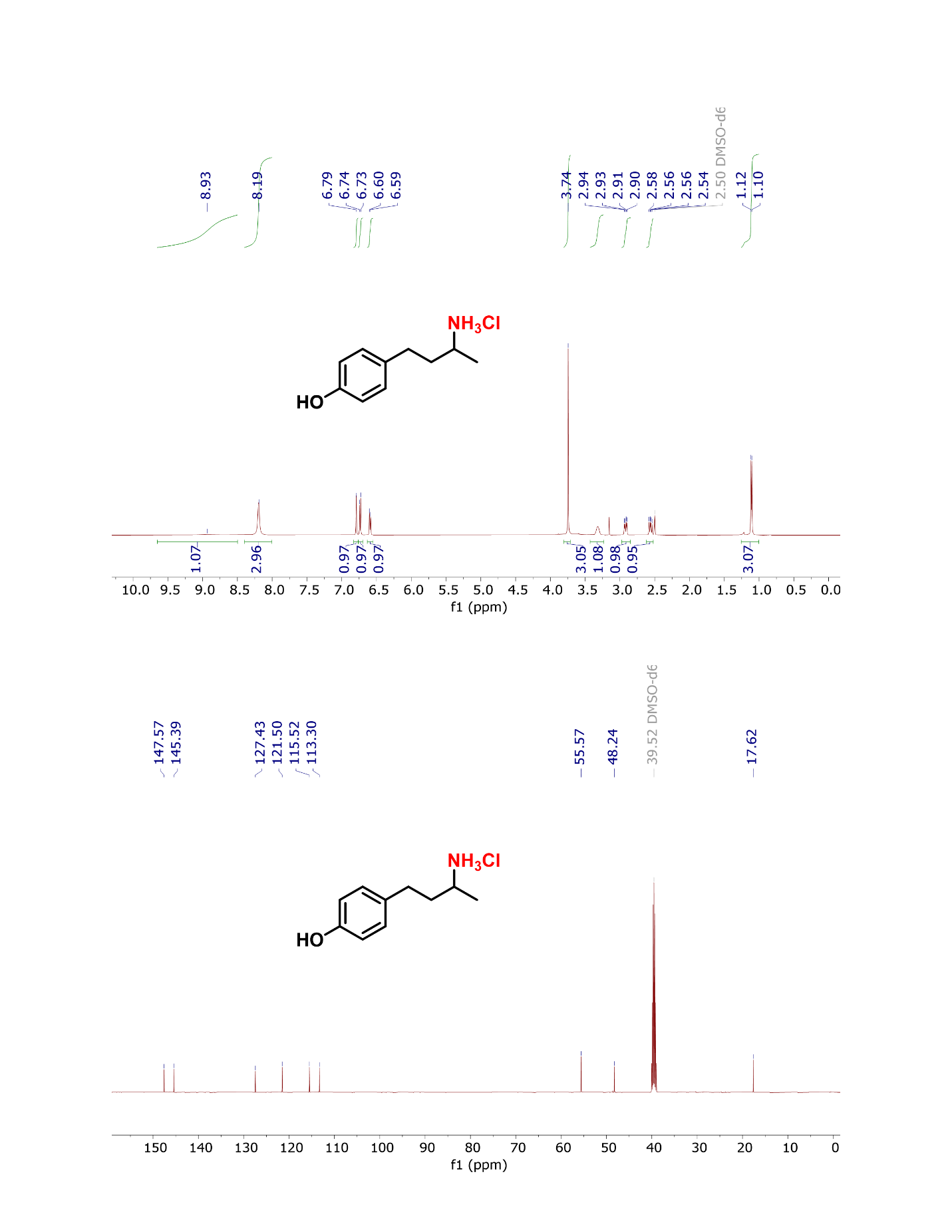


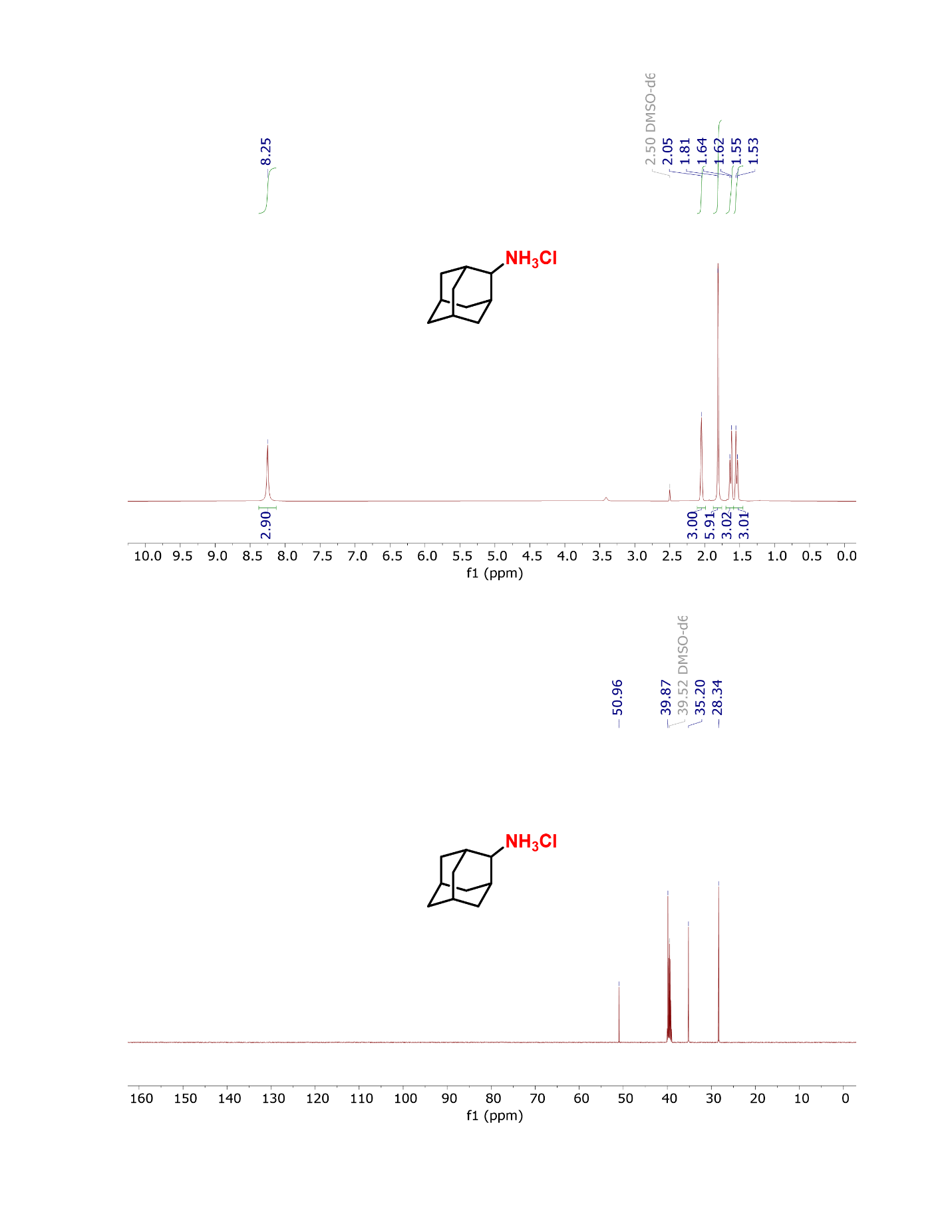


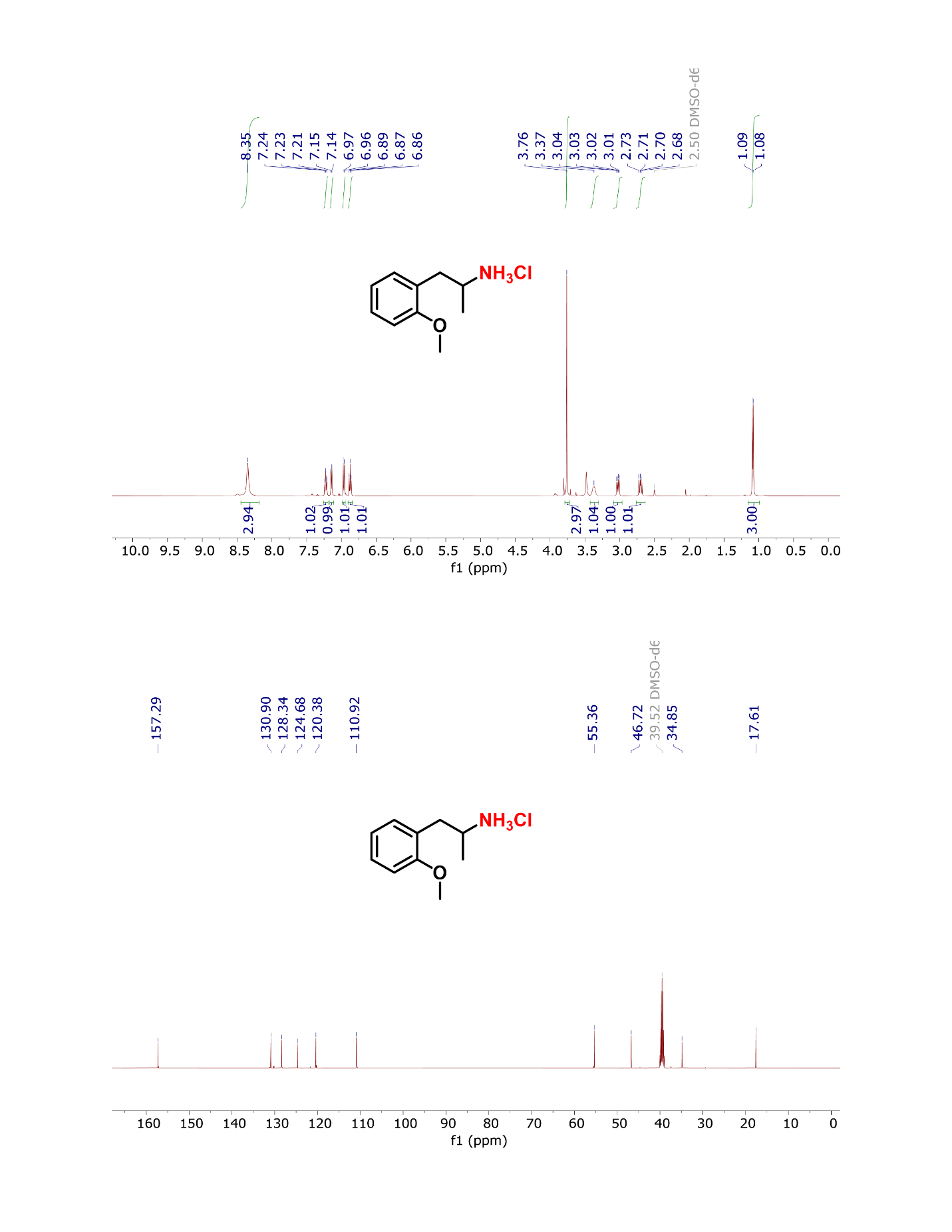


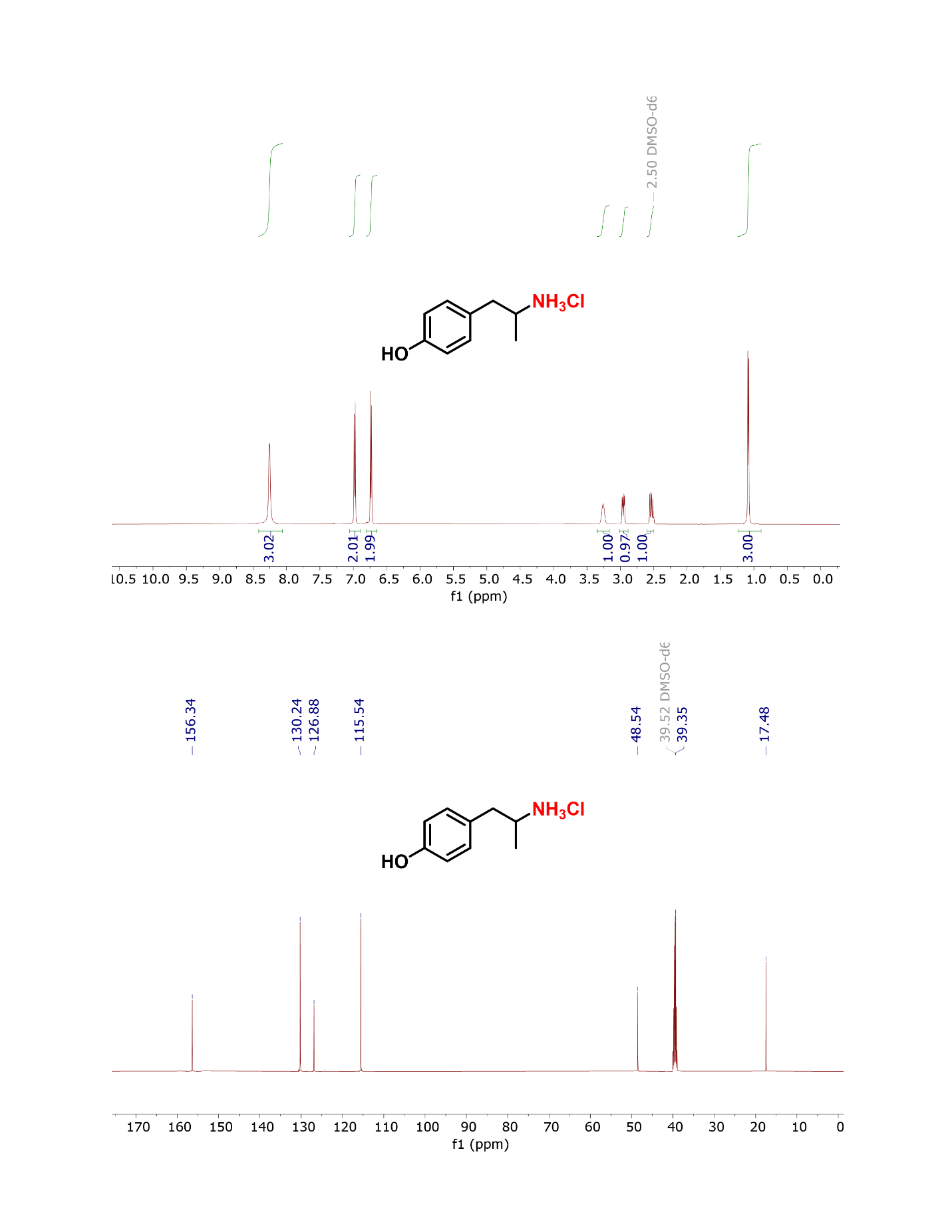


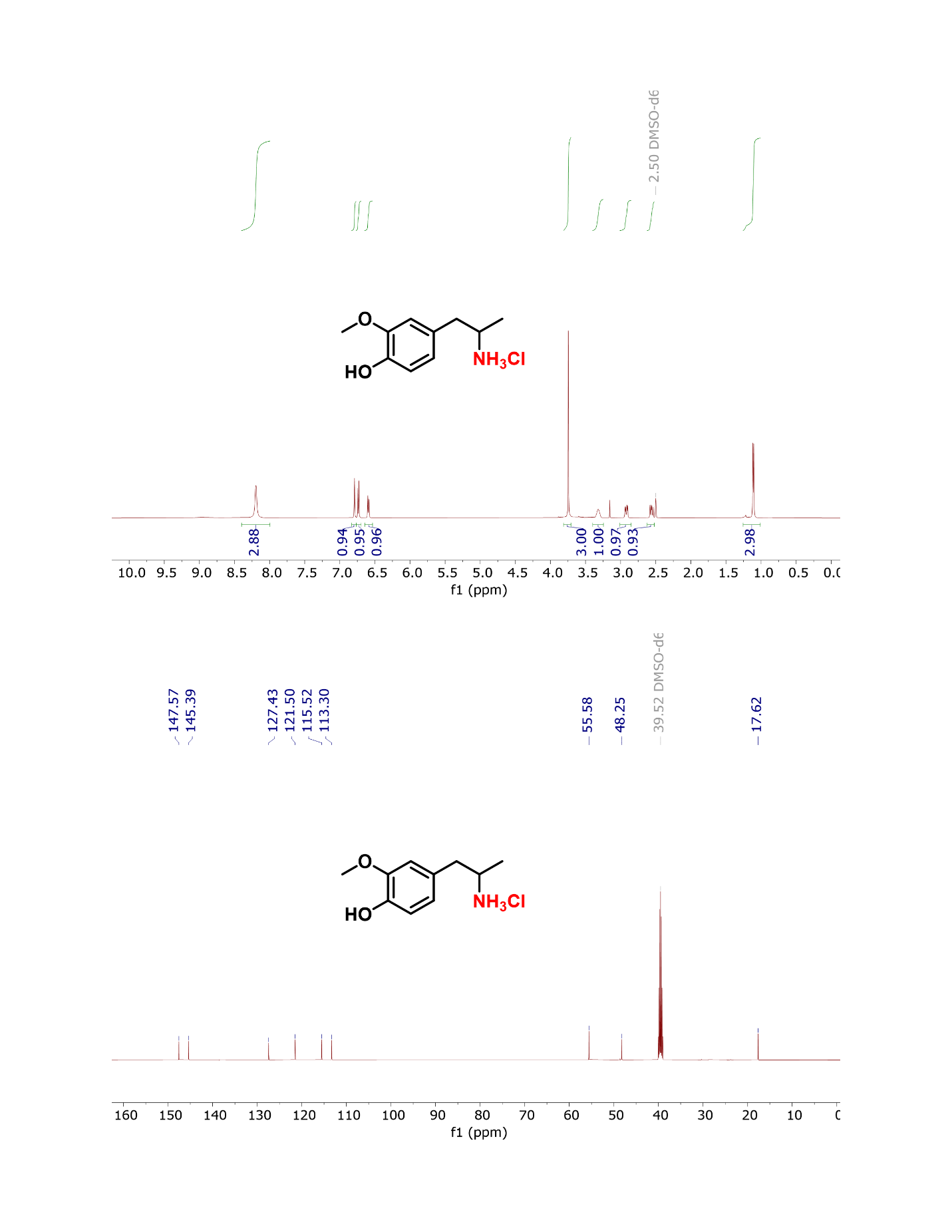

Supplement: Supplementary file 1 — Supporting Information [file ANIE-64-e19641-s001.docx]
